# Survey on Adsorption of Low Molecular Weight Compounds in Cu-BTC Metal–Organic Framework: Experimental Results and Thermodynamic Modeling

**DOI:** 10.3390/ijms23169406

**Published:** 2022-08-20

**Authors:** Antonio Baldanza, Domenico Mallamace, Giuseppe Mensitieri, Cosimo Brondi, Pellegrino Musto, Giuseppe Scherillo

**Affiliations:** 1 Department of Chemical, Materials and Production Engineering, University of Napoles Federico II, P.le Tecchio 80, 80125 Naples, Italy; 2 Departments of ChiBioFarAm—Section of Industrial Chemistry, University of Messina, CASPE-INSTM, V.le F. Stagno d’Alcontres 31, 98166 Messina, Italy; 3 Institute for Polymers, Composites and Biomaterials, National Research Council of Italy, Via Campi Flegrei 34, 80078 Pozzuoli, Italy

**Keywords:** metal–organic framework, Cu-BTC, gas adsorption, vapor adsorption, RALF model, FTIR

## Abstract

This contribution aims at providing a critical overview of experimental results for the sorption of low molecular weight compounds in the Cu-BTC Metal–Organic Framework (MOF) and of their interpretation using available and new, specifically developed, theoretical approaches. First, a literature review of experimental results for the sorption of gases and vapors is presented, with particular focus on the results obtained from vibrational spectroscopy techniques. Then, an overview of theoretical models available in the literature is presented starting from semiempirical theoretical approaches suitable to interpret the adsorption thermodynamics of gases and vapors in Cu-BTC. A more detailed description is provided of a recently proposed Lattice Fluid approach, the Rigid Adsorbent Lattice Fluid (RALF) model. In addition, to deal with the cases where specific self- and cross-interactions (e.g., H-bonding, Lewis acid/Lewis base interactions) play a role, a modification of the RALF model, i.e., the RALFHB model, is introduced here for the first time. An extension of both RALF and RALFHB is also presented to cope with the cases in which the heterogeneity of the rigid adsorbent displaying a different kind of adsorbent cages is of relevance, as it occurs for the adsorption of some low molecular weight substances in Cu-BTC MOF.

## 1. Introduction

Metal–organic frameworks (MOFs) are a class of hybrid crystalline materials composed of transition metal divalent (Zn^2+^, Cu^2+^, Mg^2+^, etc.) or trivalent (Cr^2+^, Al^2+^, Fe^2+^, etc.) cations joined together by organic ligands (phosphonates, carboxylates, or sulfonates) via strong coordinative bonds. Due to their structures, MOFs are characterized, as compared to activated carbon and zeolites, by significantly higher surface areas and a perfectly ordered molecular arrangement with pore sizes between 3 and 35 Å [1]. Different distributions of pore size and shape as well as functionalities can be achieved by simply selecting the metal center and/or the ligand [1]. This unique feature allows these materials to be used in numerous applications [2], ranging from storage media [3] and adsorbents for separation processes [4,5] to drug delivery carriers and catalysts [6,7,8,9]. In this perspective, H_2_, CH_4_, and CO_2_ are gases of particular interest due to their environmental and economic importance. H_2_ is a promising energy carrier for the substitution of liquid fuel resources applied in the automobile sector, being environmentally friendly and a fully renewable energy source [10]. CO_2_ is emitted from the combustion of fossil fuels and its emissions give a major contribution to the greenhouse effect and thus to climate change [11]. In this context, it has been observed that many MOFs have CO_2_ storage capacities [12,13]. Moreover, these materials also represent an important alternative for the separation process of the mixture CO_2_/CH_4_ [14]. In fact, the contamination of CH_4_ with CO_2_ from various sources, such as natural gas and landfill gas, leads to a decrease in the energy density and causes equipment corrosion [15]. In this regard, it was shown that a Co(II)-based MOF can preferentially adsorb CO_2_ over CH_4_, leading to a great capability of gas storage and separation [16].

Despite the exceptional flexibility of MOFs, the cation–ligand coordinative bond is susceptible to temperature and to the presence of small molecules adsorbed onto the sites of the network [17]. In extreme cases, the structure may even collapse upon high-temperature processes and/or solvent removal after the synthesis procedure, leading in some specific cases to narrowed or widened pore sizes [18]. High-temperature treatments (HTT) are often required to remove H_2_O molecules (the so-called activation) that are embedded within the MOF structure through coordinative- or hydrogen-bonding because of sorption from environmental moisture [19,20,21].

**Figure 1:**
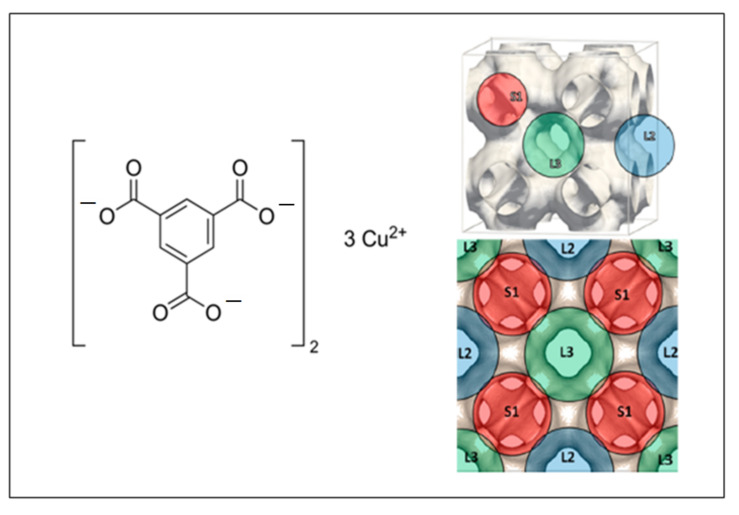
(**Left**) Molecular formula of Cu-BTC unit. (**Right**) Three-dimensional view (top) and front view (bottom) of the Cu-BTC unit cell with pockets S1 and cages L2 and L3. The right side of the figure is reprinted with permission from [30]. Copyright 2013 American Chemical Society.

It is generally known that Cu-containing MOFs are water stable and thus represent a more interesting choice from a technological perspective. In particular, HKUST-1, also referred to as Cu-BTC or Basolite^®^ C300, is one of the few commercially available frameworks and is among the most temperature/water-resistant MOFs. Since it was first documented by Chui et al. in 1999 [22], this material has been proposed in numerous applications such as gas storage [3,23,24], adsorbent for the separation of gas mixtures [25,26], molecular sensing [27], and applications as a catalyst [24,28,29]. Cu-BTC presents a three-dimensional porous framework formed by the coordination of copper cations (Cu^2+^) and benzene-1,3,5-tricarboxylate (BTC) linker molecules which form the dimeric copper paddle-wheel structural building blocks (see Figure 1, left side) [30]. The porous structure can be characterized as a combination of small pockets and larger cages [8,31,32,33]. The former type is an octahedral pocket (S1), defined by four organic apolar linkers. Among these pockets, there are two types of large cages: a first polar cage (L3) with the dimeric copper vectors pointing inward to the unit and then exposing the coordinatively unsaturated metal sites (CUMS) inside these cages; the second cage (L2) is very similar to L3, but more apolar as the CUMS are not available inside these cages [34,35,36]. As it can be observed from Figure 1 (right side), the L2 cages connect up to six L3 cages and are not connected to the small S1 pockets. To summarize, a Cu-BTC unit cell contains eight S1 pockets and four of both large L2 and L3 cages.

In the present survey, the attention is focused on the adsorption of low molecular weight compounds in Cu-BTC. The objectives are to critically review available experimental results from the literature with a special consideration for approaches exploiting the wealth of information obtainable from vibrational spectroscopy and to extensively discuss the theoretical models developed in the literature to interpret sorption data in MOFs. In addition, a modified version of an available model based on a lattice fluid approach is introduced and tested against experimental sorption isotherms of several low molecular weight compounds in Cu-BTC. 

### 1.1. Experimental Literature Data

Table 1 reports a compendium of the most significant experimental works available in the literature on this topic. It can be observed that, in the past two decades, various reports have been focused on the adsorption capacities of small apolar gases in Cu-BTC, such as noble gases, N_2_, H_2_, CH_4_, and CO_2_. Only a few reports regarding the adsorption of small polar alcohols have been published. This is particularly notable for methanol since it is one of the most abundant organic compounds in the atmosphere and, therefore, its removal in the atmosphere is of great significance [37].

### 1.2. Vibrational Spectroscopy and Other Spectroscopic Techniques

One of the main tools to investigate MOFs at the molecular level is vibrational spectroscopy (e.g., FTIR, Raman, ultrafast 2D-IR) [73]. Regarding MOFs, several vibration frequencies can be assigned to the different constituents of the framework or the guest molecules that interact with the sites belonging to the different cages. Understanding the nature of these interactions is crucial to tailor and improve MOFs properties. Structural changes of the host and/or guest molecules (e.g., adsorbed gas molecules or catalytic reactions) can be attributed and assigned to specific signal intensity changes and/or observed frequency shifts [74]. These occurrences indicate possible interactions or changes within the MOF framework. However, a correct and accurate assignment of the vibrational modes represents a challenge in vibrational spectroscopy [74]. In fact, several experimental studies making use of vibrational spectroscopy are often accompanied by theoretical interpretations, employing first-principles simulations [74,75,76] since combining theory with experiments provides complementary information for a deeper molecular characterization.

IR spectroscopy is processed through Fourier transform (FTIR) and, as its main advantage, allows for a combined solid-state technique and sampling flexibility. For instance, collection modes in FTIR such as Fourier Transform Attenuated Total Reflection (FT-ATR) and Diffuse Reflection Infrared Fourier Transform (DRIFT) do not require any sample preparation. Transmission FTIR spectra can be collected on a range of solid-state samples such as pressed pellets and thin films, while DRIFT only allows for the measurement of fine powders.

These techniques are very sensitive to intermolecular interactions, such as hydrogen-bonding (H-bonding) between water and host materials, allowing for the identification and quantification of the different molecular aggregates that are being formed [77,78]. The formation of strong H-bonds can lead to extended networks that severely affect fundamental processes such as hydration processes [79], chemical synthesis and reactions [80,81], heat dissipation [82,83], and macroscopic structure formations [83,84]. To this regard, it is essential to provide an accurate description regarding the formation of the water H-bonding network in MOFs, that can unambiguously discriminate between the framework–water and water–water interactions. For instance, it is noteworthy to mention the study conducted by Singha et al. [85] in which the authors highlighted the complex mechanism that regulates the water adsorption on MIL-53(Al) by means of DRIFT. Characteristic peaks of the OH-stretching vibration were related to water interactions with the adsorbent sites: at lower hydration levels the water interacted tightly with multiple sites, while at higher hydration levels the water interacted with fewer sites. Another study conducted on a Co2Cl2BTDD by DRIFT [78] elucidated the adsorption mechanism as a function of relative humidity (RH). It was found that water strongly binds with the open Co^2+^ sites of the framework to subsequently form one-dimensional chains of H-bonded molecules that develop bridges between the Co^2+^ sites. Upon an increase in RH, the water chains filled the pores of the network and occupied the entire pore volume before RH attained 30%.

In the context of Cu-BTC MOF, different works have been performed to elucidate the interactions of guest species with the framework. It is noteworthy to note the spectroscopic study regarding the adsorbate–adsorbent interaction in HKUST-1 performed by Bordiga et al. [23,40]. In their first work regarding this topic [40], the authors investigated the dehydration process (activation) by means of the XRD, UV−Vis, EXAFS, XANES, and Raman spectroscopies. They experimentally showed that the removal of coordinated water molecules, chemically bound to the Cu^2+^ sites, led to an unchanged oxidation state of copper, a preserved crystalline nature of the material, and promoted the reduction in the cell volume due to the shrinking of the [Cu_2_C_4_O_8_] cage. In the dehydrated state, they observed the formation of labile Cu^2+^···CO and Cu^2+^···H_2_ adducts, detecting for the first time the signal of Cu(II) carbonyl and dihydrogen complexes formed inside a crystalline microporous hosting matrix. In a follow-up study, Bordiga et al. [23] improved the preparation method for the Cu-BTC synthesis and performed IR spectroscopic measurements in transmission mode using a properly designed cryogenic cell, assessing the interaction of HKUST-1 sites with several adsorbates, such as NO, CO_2_, CO, N_2_, and H_2_. Interestingly, the interaction of CO_2_, CO, and N_2_ allowed to distinguish between a first type of Cu^2+^ sites located at the external faces of the crystals and a second type of Cu^2+^ sites regularly contained within the cages of the framework.

IR and Raman spectroscopy are complementary techniques with different selection rules and are often implemented jointly to study the adsorption of gases into MOFs. Raman spectroscopy is based on the detection of photons that are inelastically scattered from the sample under observation when it interacts with the radiation of a single frequency laser [85]. As the laser frequency is shifted up or down due to the interaction of the molecular vibrations, the produced spectral lines (fingerprint) correspond to the different vibrational modes of the sample material. Raman spectroscopy presents the main advantage, as compared to FTIR spectroscopy, of being able to collect high-resolution vibrational spectra at very low wavenumbers (ca. 10 cm^−1^). To this regard, metal containing materials are characterized by low frequency modes, such as metal–ligand stretches [85]. In general, Raman spectroscopy methods allow for the monitoring of solid-state samples such as pressed powders, thin films, and suspensions. However, the major limit for these techniques when studying the interactions between MOFs and adsorbates is the sample fluorescence as even weak fluorescent backgrounds can overcome Raman signals. In fact, several MOF samples that contain organic building blocks can lead to fluorescence phenomena that can mask the Raman signal [85]. In this sense, Resonance Raman (RR) may not be suitable since it relies on sources at frequencies near to those of a molecule’s electronic transition [86]. For this reason, Raman spectroscopy is suitable for MOF materials with no characteristic fluorescence [85].

Raman spectroscopy has been widely used to investigate the hydrolyzation and water stability of MOF networks [40,87,88]. Notably, for Cu-BTC frameworks, in [88], the authors reported an experimental investigation by means of Raman spectroscopy on the decomposition process of Cu-BTC exposed to air moisture at 300 K and 70% of RH. Raman measurements indicated structural deterioration of the framework due to hydrolysis which affected a significant fraction of the Cu-O bonds of the crystal. This occurrence led to an irreversible process for exposure times longer than 20 days. Raman spectra revealed a shifting of the peak positions and variable intensities of the main Raman bands of the material, attributed to Cu-Cu, Cu-O, and O-C-O stretching modes. Interestingly, the coexistence of two types of paddle-wheels with different structures was observed, corresponding to hydrolyzed and nonhydrolyzed paddle-wheels, as confirmed by the detection of the Raman peak split attributed to the Cu-Cu vibration in two well distinguishable components.

Other significant techniques suitable to monitor and characterize the MOF structure are optical electronic spectroscopy and X-ray spectroscopy. Optical electronic spectroscopy allows for the detection of electronic energy levels and bonding features of different molecules and materials [89,90]. Structural frameworks formed by transition metal-based complexes and coordination polymers, such as MOFs, can exhibit a large range of electronic behaviors (from semiconductor to conductor depending on the framework structure) and, therefore, their electron transitions fall in the UV-visible and Near Infrared (NIR) wavelength range [89,90]. Therefore, optical spectroscopy methods, such as absorption and emission spectroscopy, are suitable to investigate the electronic structure of MOFs. These techniques allow for the treating of homogenous microcrystalline powders and are easily tunable for in situ measurements at controlled environmental conditions.

X-ray spectroscopy allows for the analysis of electron transitions upon the absorption or emission of X-ray photons [91,92]. Based on an excited state induced by the energy of a photon, an atom moves to a higher energy level and then returns to its “unexcited” energy level. These energy transitions translate to the emission of photons with a wavelength characteristic of the sample material under observation [91,92]. In recent years, regarding MOFs, the use of techniques based on the X-ray absorption spectroscopy (XAS) has spread. That is, X-ray absorption near edge structure (XANES) and extended X-ray absorption fine structure (EXAFS) allow for the analysis of atomic distances, the coordination geometry, and oxidation state of a specific metal element, making them suitable to collect data regarding the MOF structural changes and the host–guest interactions. Similarly, in this case, these techniques deal with solid-state samples and allow in situ monitoring of the adsorption processes. 

Reference to several significant works based on vibrational spectroscopy conducted on Cu-BTC networks and aimed at elucidating their interactions with adsorbates is provided in Table 2.

### 1.3. Theoretical Modeling and Simulation Approaches for Sorption of Low Molecular Weight Compounds in MOFs

Vibrational spectroscopy is often assisted by theoretically calculated frequencies that can help in assigning the spectroscopic bands of interest. One simple method consists of the normal coordinate analysis that is usually used to provide a harmonic vibrational frequency [95,96,97,98]. However, this method has often been inconsistent with the assignment of the spectral bands of complex materials such as MOFs [98]. In this respect, a first alternative, preferred by many researchers, consists of the empirical modeling that provides a quick and effortless evaluation of the adsorption phenomena.

The Langmuir–Freundlich (LF) isotherm, widely known also as Sips isotherms [99], is a semiempirical three-parameter model that includes mathematical features of both the Langmuir and Freundlich isotherms. Although the thermodynamic consistency of this model exhibits flaws in the regions of very low pressure (since it does not recover the Henry’s law limit), the simple form of the equation allows for the modeling of either subcritical or supercritical isotherms, without requiring for the definition of the saturation pressure for the adsorbate. The equation is the following [50]:

(1) $$q=q^{max}\cdot \frac{{\left(b\cdot p\right)}^{1/n}}{1+{\left(b\cdot p\right)}^{1/n}}$$

where *q* represents the adsorbed amount, *p* is the pressure, *q^max^* is the maximum adsorption capacity, *b* is the affinity constant, and *n* is the heterogeneity coefficient, respectively. In particular, for *n* = 1, the Sips isotherm reduces to the classic Langmuir isotherm, applicable to homogeneous adsorbent–adsorbate systems. Sips parameters are dependent on the temperature [100] but, typically, *q^max^* and *n* are considered independent from the temperature to keep the model application simpler. In the work performed by Aprea et al. [50], the Sips model was adopted to model the CO_2_ adsorption isotherms on a laboratory-synthesized Cu-BTC framework at several temperatures (283, 293, 318, and 343 K) and for pressures up to 0.1 MPa. Although it was possible to gather some preliminary data regarding the Cu-BTC saturation capacity and a homogenous-type (Langmuir) adsorption system, this simple model did not allow for the retrieval of information regarding the nature of the adsorbate–adsorbent interaction. 

Under this perspective, the Virial–Langmuir (VL) model allows for the gathering of some information regarding the nature of the adsorbate–adsorbent interaction. This model consists of the addition of two virial coefficients to the Henry constant and naturally recovers Henry’s law in the low concentration limit [101]. The equation is expressed as follows [45,54]:

(2) $$p=\frac{q^{max}\cdot q}{\beta \cdot \left(q^{max}-q\right)}\cdot \exp\left(b\cdot q+c\cdot q^{2}\right)$$

where *β* is the Henry constant, and *b* and *c* are the second and third virial coefficients, respectively. For instance, in [54], authors conducted a comparative adsorption study of three gases, such as CO, CO_2_, and CH_4_, on two adsorbents, namely Cu-BTC (or HKUST-1) and Cr-BDC (or MIL-101). The gravimetric adsorption equilibrium measurements on the samples were performed using a magnetic suspension balance at three different temperatures (295, 318, and 353 K) and pressures ranging from 0 to 10 MPa. In this instance, the use of the model allowed the authors to firstly evaluate the enthalpy of adsorption at zero loading and the enthalpy variation with loading at 295 K. In the former case, the enthalpy of adsorption of the three gases only resulted in small differences, due to coordinatively unsaturated metal centers present in the Cu-BTC framework [49] that were either not open or hindered by the presence of solvent molecules left over from the synthesis procedure; in the latter case, both CH_4_ and CO_2_ showed a slight variation in enthalpy with loading, while CO only showed a considerable decrease in enthalpy of adsorption. This occurrence was attributed to electrostatic interactions that dominate only the low loading region, while as the sites available for the interaction are progressively filled up, the enthalpy of adsorption drops down sharply. From the adsorption isotherm data, it was also possible to observe that CO_2_ exhibited the highest capacity, while CH_4_ had a lower capacity than CO in the low-pressure region and then progressively exceeded it in the high-pressure region. The initial behavior of the comparison between CH_4_ and CO was also confirmed by Henry’s constant (evaluated from the VL model), that is higher for CO than for CH_4_ at 295 K due to its dipole moment. However, as interaction sites are progressively filled up, initial electrostatic interactions are overcome by dispersion interactions and the larger polarizability of CH_4_ results in its higher capacity.

Empirical modeling has provided a theoretical framework not only for the guest–host interaction, but for studying multicomponent adsorption mechanisms as well. To this aim, the Dubinin–Astakhov (DA) model is widely used for modeling the single-component isotherms and provides a fair prediction of the multicomponent isotherms [102]. As opposed to the Langmuir adsorption isotherm, this model is related to the micropore volume filling without the formation of successive surface layers [102]. The equation is expressed as follows [102]:

(3) $$q=q^{max}\cdot exp\left[-{\left(\frac{R\cdot T}{\varepsilon }\cdot ln\frac{p_{s}}{p}\right)}^{n}\right]$$

where *R* is the ideal gas constant, *T* is the temperature, *ε* is the characteristic energy of adsorption, *p_s_* is the saturation pressure, and *n* is the heterogeneity parameter, respectively. The introduction of the parameter *n* allows us to account for the surface heterogeneity typical of microporous adsorbents such as MOFs. In their work [63], Gomez et al. used the DA model for a non-linear regression of single-component adsorption isotherms measured experimentally at 298 K and in a pressure range of 0–5 MPa. Based on that, the authors were then able to predict the adsorption isotherms of binary and ternary mixtures containing CO_2_, CH_4_, and N_2_ within Cu-BTC frameworks.

Although empirical models are readily implemented to retrieve data on the adsorbent–adsorbate interaction, such as the isosteric heat of adsorption, results are not always consistent with experimental observations [35,43,49,51,57,65,69,70,93,103]. To provide a robust background on the mechanisms taking place during the gas adsorption on MOF materials and an interpretation of their relative vibrational spectra, over the last decade different ab initio computational methods have been implemented. For Cu-BTC frameworks, large efforts have been made in this direction using both density functional theory (DFT) [49,57,65,103] and Grand Canonical Monte Carlo (GCMC) calculations [35,43,51,69,70,93]. Typically, a large computational effort is required for these calculations as the structure of MOFs comprise large crystal cells. Therefore, the use of these approaches is generally limited to small areas, such as clusters, or is combined with the use of molecular dynamics (MD) as well.

DFT methods are usually preferred as they possess a relatively higher computational efficiency and present an acceptable accuracy in predicting the vibrational spectra with their relative band intensities and frequencies [96]. A major drawback of DFT methods is the incapability of calculating weak van der Waals forces and, hence, methods such as dispersion-corrected DFT (DFT-D) and van der Waals-DFT are used to account for this discrepancy [95]. Conversely, MD simulations of MOFs rely on the development of force fields (FFs) that can describe the molecular interaction between the framework and the guest molecules. Commonly used Force Fields (FFs) for MOFs are the general Amber force field (GAFF) [104] and the universal force field (UFF) [105] that allow us to accurately model the organic links, but are less effective in describing the coordination and the geometry of the metal center [106]. To address this issue, FF extension for MOFs has also been developed, i.e., the MOF-FF [107] and the Quick-FF [108].

Concerning the Cu-BTC frameworks, some works warrant mention. For instance, Supronowicz et al. [74] studied the interactions of CO, CO_2_, OCS, SO_2_, NO, NO_2_, N_2_O, NH_3_, PH_3_, and other small molecules with the undercoordinated metal centers of the HKUST-1, by means of the DFT. Authors retrieved the adsorption energies on the Cu^2+^ sites of the paddle-wheel and found the following ranking: NH_3_ > H_2_O > PH_3_ > H_2_S > SO_2_ > CO ∼ OCS ∼ CO_2_ ∼ N_y_O_x_ > N_2_ > O_2_. They classified the observed interactions into three categories: (1) weak physisorption, (2) polarization and electrostatics, and (3) strong acid–base.

García-Pérez et al. [47] investigated the adsorption of several quadrupolar and non-polar gases on Cu-BTC by means of combined adsorption isotherms and Monte Carlo (MC) simulations. In this study, the authors could identify four main adsorption sites: site I close to the copper atoms, site I′ within the larger cavities, site II located in the center of the smaller octahedral cages, and site III at the windows of the four open faces of the octahedral cage. Monte Carlo simulations allowed us to detect the octahedral cages (sites II and III) and the big cages (site I′) as the preferred positions for adsorption, while in the case of site I (near the copper atoms), sites remain empty over the entire range of analyzed pressures, possibly due to the reduced accessibility of these sites. Interestingly, the occupation of the different sites by CH_4_ and C_2_H_6_ exhibited small differences as compared to O_2_ and N_2_; this finding being attributed to the quadrupole moment of the polar molecules. While CH_4_ molecules predominantly occupied the sites of type II, the N_2_ occupied both I′ and II an equivalent amount. The molecular sitting for O_2_ showed an intermediate behavior between those observed for CH_4_ and N_2_.

In their work, Van Assche et al. [58] studied the adsorption of various polar adsorbates (such as methanol, ethanol, 1-propanol, 2-propanol, 1-butanol, 1-hexanol, water, acetone, acetonitrile, tetrahydrofuran, and N,N-dimethylformamide) as well as apolar adsorbates (such as n-hexane, n-heptane, and m-xylene cyclohexane) on Cu-BTC frameworks. The authors observed that alcohols characterized by a longer carbon chain (and thus less polar) have higher uptakes at lower vapor pressures. Interestingly, a significant two-step uptake was noticed for smaller alcohols in the measured vapor pressure range, the effect being more remarkable for methanol and ethanol. Regarding the alkanes, these molecules filled up the sites of the Cu-BTC structure at low vapor pressures due to their favorable interaction with the host structure. Despite the strong interaction of polar adsorbates with the Cu-BTC structure, the material also showed a quite apolar nature due to the presence of the aromatic counterpart. This behavior was also observed when performing GCMC simulations at 313–343 K for methanol using the Cu-BTC crystal structure proposed by Chui et al. [22]. 

Listed in Table 2 is a series of combined experimental and theoretical approaches used to characterize the interaction between the adsorbent and adsorbate, retrieved from the literature.

All the described semiempirical thermodynamics models, as well as other phenomenological approaches, which have been extensively reviewed by Brandani [109], suffer from a lack of thermodynamic consistency in dealing with adsorbates molecules with an appreciable difference in size and do not account in a full predictive fashion for the adsorbate–adsorbate and/or adsorbate–adsorbent interactions on the basis of the pure component properties [109]. Despite these semiempirical models being able to exhibit a good fitting capability in view of the large number of phenomenological fitting parameters, they are not suitable for a full predictive approach since these adjustable parameters are not rooted in a rigorous physical background, so their safe use is limited to the condition of the experimental data adopted for the non-linear regression.

To overcome this drawback, a lattice fluid equation of state theory firmly rooted on a statistical thermodynamics background [109,110], aimed at modeling the adsorption thermodynamics of multicomponent fluid mixtures within a rigid adsorbent has been recently proposed in the literature. This approach, known as the Rigid Adsorbent Lattice Fluid model (RALF), has been successfully applied to mixtures of gases and/or vapors within rigid zeolites and MOF systems. To this regard, in the present contribution, we have implemented for the first time the RALF model to investigate the adsorption of pure CO_2_ in Cu-BTC. Moreover, in the present investigation we propose an extension of the original RALF model (which is intrinsically a pure mean-field theory), accounting for specific adsorbates–adsorbates and adsorbates–adsorbent interactions. This model, named RALFHB model, has been applied to investigate the adsorption of CH_3_OH in Cu-BTC. In Section 2, we report the fundamentals of the RALF and RALFHB models.

## 2. Lattice Fluid Thermodynamics Models

### 2.1. RALF Model

The Rigid Adsorbent Lattice Fluid (RALF) model has been developed by Brandani [109,110] with the aim of describing sorption isotherms of low molecular weight fluid mixtures within a rigid solid adsorbent (such as zeolites and several types of MOFs). Brandani has recently proposed [111] an extension of this model that also accounts for the flexibility of the solid, which has been implemented in the case of a MOF adsorbent that undergoes structural changes in the presence of an adsorbate (breathing transition). In the present contribution, since such kind of structural transitions are not present in the case of binary Cu-BTC/penetrant systems, we take the assumption of rigid solid as reasonable for a quantitative description of the sorption thermodynamics. Therefore, the original version of the Brandani model (indicated hereafter as RALF) has been considered. For the sake of brevity, in the following, we only report the basic equations of this model, referring the interested reader to the original literature for the details regarding the derivation of the equations and the meaning of all the involved variables [109,110].

The RALF model represents an ad hoc extension of the original Sanchez and Lacombe (SL) multicomponent Lattice Fluid model [112,113,114], originally developed to deal with compressible fluid mixtures. The SL model accounts for only self- and cross-mean-field pair interactions and describes the compressibility of the system assuming the presence of empty sites within the lattice. To re-adapt this LF model to the case of a solid adsorbent, Brandani [109,110] assumes that the volume of this mixture V is identified as the apparent volume of the solid *V_S_* (i.e., one of the solid including its micropores) in the mixture so that the following relationships hold:

(4) $$V=V_{S}=\frac{m_{S}}{\rho_{S}}=\frac{\sum_{j=1}^{t}m_{j}}{\rho }$$

where *m_S_*, *ρ_S_*, *ρ*, *m_j_*, and *t* represent the mass of the solid, the apparent density of the solid, the density of the adsorbent phase, the mass of *j*-th component, and the total number of components in the adsorbent phase, respectively. Commonly, the Equation of State, EoS, for the mixtures formed by the adsorbent phase is not available, but it could be possible to use the EoS and/or dilatometric equilibrium data of the pure solid to circumvent the problem. Brandani [109] proposed the following relationship, valid at any temperature, *T*:

(5) $$V_{S}=V_{S}^{\infty }+\left(V_{S}^{0}-V_{S}^{\infty }\right)exp\left(-\beta_{T}P\right)+\Delta V_{S}$$

where $V_{S}^{0}$ is the equilibrium solid volume in vacuum and $V_{S}^{\infty }$ is the equilibrium solid volume approached at infinite pressure *P*, in absence of the adsorbates (i.e., pure solid apparent volume) at the given *T*. In principle, these two values can be provided by an EoS of the pure solid. $\beta_{T}$ represents the isothermal equilibrium compressibility factor at the given *T* and in many solid phases, which do not undergo allotropic transformations, can be assumed to be quite independent upon *P* (this result should be consistently confirmed by the pure solid EoS adopted). Such an approximation is applied in Equation (5). More in general, to avoid the described simplification on $\beta_{T}$, the first two terms can be lumped in a single expression $V_{S}^{pure}\left(T,P,N\right)$ directly provided by the adopted pure solid EoS (where the *i*-th component of the vector ***N*** represents the number of molecules of component *i*). Finally, $\Delta V_{S}$ represents a correction term accounting for possible solid volume rearrangements induced by the adsorbates and it is, therefore, a function of *P*, *T*, and concentration. This term is significant only in the case of flexible solid structures, so that the RALF model assumes that both $\Delta V_{S}$ and $\beta_{T}$ are equal to zero. Therefore, *V_S_(T*) is neither a function of adsorbate concentration nor of *P*; then, according to Equation (4), the volume of the mixture *V* is not provided by any EoS for the mixture and it is approximated by *V_S_*(*T*), whose value is commonly supplied by a preliminary experimental investigation of the pure solid. To this regard, a common further reasonable assumption is that the value of *V = V*_S_ is taken also to be independent of *T*.

In conclusion, based on the rigid network of the solid, it descends the main assumption that the density of the adsorbent mixture is not dictated by the EoS expression provided by the SL theory. However, it is postulated that the Gibbs energy still follows the functional dependence on the state variables of the general out-of-equilibrium constitutive class provided by the SL model. For instance, in an ***N***, *P*, *T* ensemble, these are given by *P*, *T*, *V*, and by the vector of the number of molecules of each component in the phase considered, ***N*** (hereafter, the symbol *N* stands for the scalar variable representing the total number of molecules of the phase of interest). Brandani recognizes that this approach represents formally the basis of the Non-Equilibrium Lattice Fluid (NELF) model previously proposed by Sarti and Doghieri [115,116,117]. In fact, the NELF model is an extension of the SL model, in the framework of thermodynamics with internal state variables [118,119,120], to deal with sorption thermodynamics in polymers kinetically frozen in an out-of-equilibrium glassy state. It is worth noting that, in the case of glassy polymers, the frozen value of the mixture volume depends on the non-equilibrium thermomechanical history of the pure polymer sample up to the start of the sorption test and, hence, it can significantly differ (it is commonly higher) from the corresponding equilibrium value at the same *P* and *T* [115,116,117]. Conversely, the described simplifications in Equation (5) of the RALF model are rooted in classical equilibrium thermodynamics and the non-equilibrium value of *V_S_* = *V* must be properly intended as an “average” value of the true equilibrium value in the range of *T*, *P*, and concentration of interest so that, under the rigid solid assumption, it is expected to be close to the true equilibrium value of the mixture. However, in the development of both the RALF and NELF models, the different rationale that stays behind the assumption of a frozen *V* is not relevant, since the only significant hypothesis is given by the use of the same functional form of the Gibbs energy provided by the SL framework.

In the SL model and, hence, in the RALF model, $N_{j}$ and $r_{j}$ represent the number of molecules and the lattice sites occupied by a molecule of species *j*, respectively. Moreover, $N_{0}$ represents the number of vacancies (the vacancies are assumed to simply occupy one empty site, so that *r*_0_ is assumed to be equal to 1). Therefore, if $v^{*}$ is the cell volume within the lattice, the total volume *V* of the mixture formed by *t* species (including the solid in the case of the adsorbent phase of RALF model) is given by:

(6) $$V=\left(N_{0}+rN\right)v^{*}=\left(N_{0}+\overset{t}{\underset{j=1}{\sum }}N_{j}r_{j}\right)v^{*}$$

The close-packed volume of the mixture $\left(V^{*}\right)$ is obtained by setting $N_{0}=0$ in Equation (6). Therefore, the reduced volume $\left(\tilde{v}\right)$ of the mixture is given by:

(7) $$\tilde{v}=\frac{1}{\tilde{\rho }}=\frac{\rho^{*}}{\rho }=\frac{V}{V^{*}}=\frac{\left(N_{0}+\sum_{j=1}^{t}N_{j}r_{j}\right)v^{*}}{rNv^{*}}=\frac{N_{0}+\sum_{j=1}^{t}N_{j}r_{j}}{rN}$$

In Equation (7), ρ represents the actual density of the mixture and $\rho^{*}$ the density of the mixture in the close-packed condition. The volumetric fraction of the *j*-th species and of the LF empty sites are, respectively (hereafter, superscript *L* stands for lattice fluid including the empty sites):

(8) $$\varphi_{j}^{L}=\frac{N_{j}r_{j}}{N_{0}+rN}\;\;\mathrm{and}\;\varphi_{0}^{L}=1-\sum_{j=1}^{t}\frac{N_{j}r_{j}}{N_{0}+rN}$$

A first issue related to the use of the SL arises in the need to define a mixing rule for $v^{*}$. To this regard, in the original SL model, the authors [112,113,114] proposed a mixing rule which allows us to retain the close-packed molecular volume proper of the pure state also within the mixture for each component:

(9) $$r_{j}v^{*}=r_{j}^{0}v_{j}^{*}\;\;\mathrm{for}\;\mathrm{each}\;j=1,\;2,\;\ldots ,\;t$$

In Equation (9), $r_{j}^{0}$ represents the number of mers (i.e., of cells) occupied by a molecule of the species *j*-th in its pure phase (superscript 0 refers to the pure component). By summing upon the *t* species, it is evident that Equation (9) implies that the close-packed volume of the mixture is additive in terms of the close-packed volume of the pure species. Equation (9) introduce an *r_j_* term for each species so that there are *t* equations but *t* + 1 mixture variables (including $\;v^{*}$) need to be determined. 

To close the problem, in the same series of papers [112,113,114], the authors assumed that the total number of LF cells occupied within the mixture is additive with respect to the LF representation of pure components so that the following equation holds:

(10) $$\overset{t}{\underset{j=1}{\sum }}r_{j}^{0}N_{j}=\overset{t}{\underset{j=1}{\sum }}r_{j}N_{j}=rN=N\overset{t}{\underset{j=1}{\sum }}r_{j}x_{j}$$

where $x_{j}$ represents the molar fraction of *j*-th component. From Equation (10) it follows that:

(11) $$r=\frac{\sum_{j=1}^{t}r_{j}^{0}N_{j}}{N}=\overset{t}{\underset{j=1}{\sum }}r_{j}^{0}x_{j}$$

Equation (11) represents the searched operative expressions for the “average” mixture parameter *r* only as a function of the corresponding pure component parameters and concentration. According to Equations (9) and (10), the close-packed volume is given by:

(12) $$V^{*}=\overset{t}{\underset{j=1}{\sum }}r_{j}N_{j}v^{*}=\overset{t}{\underset{j=1}{\sum }}r_{j}^{0}N_{j}v_{j}^{*}=rNv^{*}$$

Equation (12) provides the additional function which allows us to close the mixing rules conditions along with Equation (9). From Equations (9)–(11), the following expression is obtained [112,113,114]:

(13) $$v^{*}=\overset{t}{\underset{j=1}{\sum }}\varphi_{j}^{0}v_{J}^{*}$$

where:

(14) $$\varphi_{j}^{0}=\frac{r_{j}^{0}N_{j}}{rN}=\frac{r_{j}^{0}x_{j}}{\sum_{j=1}^{t}r_{j}^{0}x_{j}}$$

Equations (13) and (14) represent the needed operative expressions for the $v^{*}$ of the mixture as a function only of pure component parameters and concentration.

Finally, the close-packed mass density of the mixture, according to Equation (12), is provided by the following expression:

(15) $$V^{*}=\frac{\sum_{j=1}^{t}m_{j}}{\rho^{*}}=\overset{t}{\underset{j=1}{\sum }}r_{j}N_{j}v^{*}=\overset{t}{\underset{j=1}{\sum }}r_{j}^{0}N_{j}v_{j}^{*}=\overset{t}{\underset{j=1}{\sum }}\frac{m_{j}}{\rho_{j}^{*}}$$

where the last equality is provided by the following relationship for each pure component [115,116,117]:

(16) $$r_{j}^{0}v_{j}^{*}=\frac{M_{w,j}}{\rho_{j}^{*}}$$

where $M_{w,j}$ represents the molecular weight of *j*-th species. From Equation (15), the following equation is easily derived:

(17) $$\frac{1}{\rho^{*}}=\overset{t}{\underset{j=1}{\sum }}\frac{w_{j}}{\rho_{j}^{*}}$$

where $w_{j}$ represents the mass fraction of *j*-th component. Equation (17) represents the operative expression which provides the mixing rule for $\rho^{*}$ as a function of concentration and only of pure component parameters.

We remark that the same set of mixing rules provided by Equations (9) and (10) appear both in the RALF and in the NELF models. In a full predictive framework, according to the original SL model, the close-packed volume of each pure penetrant to be adopted in the case of the adsorbent phase can be assumed to be equal to the one adopted in describing the external fluid phase. However, in the case of adsorbed molecules in a rigid solid, due to confinement constraints, it is reasonable (still retaining as mixing rules the Equations (9) and (10) also in the adsorbent phase) to assume that in the solid-penetrant phase the close-packed volume of each pure component, to be adopted for the generic adsorbate, is larger than that in the pure bulk fluid phase [121]. To this regard, Brandani [109,110], in the development of the RALF model, introduced a pair mixture parameter for each penetrant $\left(\xi_{jA}\right)$, to properly re-scale the $v_{j}^{*}$ to be adopted within the adsorbent phase:

(18) $$v_{jA}^{*}=\left(1+\xi_{jA}\right)v_{j}^{*}\;\mathrm{and}\;\rho_{jA}^{*}=\frac{\rho_{j}^{*}}{1+\xi_{jA}}$$

In Equation (18), the subscript *jA* stands for the couple made by the *j*-th component (different from the solid adsorbent) and by the solid itself (*A* stands for the adsorbent) and, according to this approach, the parameter $\xi_{jA}$ is expected to be non-negative and, for a given solid adsorbent, to be larger for penetrant molecules with a larger kinetic diameter.

Along with the described procedure for $v_{j}^{*}$, Brandani proposed, according to Equation (18), to re-scale the close-packed density of the component *j*, $\rho_{j}^{*}$, whose value is consistently expected to assume a lower value in the case of the adsorbent phase. In particular, the shape of Equation (18) allows us to retain the same value of$\;r_{j}^{0}$ that is used for each adsorbed molecule both in the external fluid phase and in the adsorbent phase.

This represents the main departure of the RALF model from the NELF approach but, since it does not introduce any additional concentration dependence for the mixture parameters, it does not affect the formal derivation of the RALF expressions for the chemical potentials. To this regard, the subscript *jA* can be formally replaced by the subscript *j*, just recognizing that in the adsorbent phase, $v_{j}^{*}$ and $\rho_{j}^{*}$ are intended to be the concentration independent quantities provided by Equation (12). This formal simplification is adopted by the expressions of the RALF model reported in the following discussion. By inspecting the SL expressions for the equilibrium chemical potentials, Brandani recognized that the SL model suffers from an intrinsic thermodynamic inconsistency: in multiphase phases differently from the pure case, when the volume (at a given *T* and concentration) diverges positively, the model does not recover the expression of the chemical potentials of the ideal gas state. This is a well-documented issue [122,123,124,125,126,127,128] which arises from the common set of mixing rules of $v^{*}$ adopted in the SL literature. To this regard, different approaches to correct this inconsistency have been proposed [122,123,124,125,126]. Moreover, it has been pointed out that a more complex lattice fluid model, the so-called Non-Random Hydrogen-Bonding (NRHB) model [129,130], is conceived in a way that this drawback is overcome [127,128].

The first step in the development of the RALF model, working in an ***N***, *V*, *T* ensemble, is the introduction of a posteriori correction of the expression of the combinatorial term of the SL Helmholtz energy which allows us to disgard of the mentioned inconsistency. Starting from this corrected expression, it was found [109] that (within an additive constant and terms that are only dependent upon *T* and that are not involved in the determination of the chemical potential expressions as well as of the EoS) the Gibbs energy of the SL model can be written as:

(19) $$\frac{G^{SL}}{nRT}=r\left[-\frac{\tilde{\rho }}{\hat{T\;}}+\frac{\left(1-\tilde{\rho }\right)ln\left(1-\tilde{\rho }\right)}{\tilde{\rho }}+\frac{ln\left(\tilde{\rho }\right)}{r}+\overset{t}{\underset{j=1}{\sum }}\frac{\varphi_{j}}{r_{j}}\;ln\left(\varphi_{j}\right)\right]+z$$

Starting from Equation (19), the corresponding residual Gibbs energy takes the form:

(20) $$\frac{G^{R,SL}\left(P,T,N\right)}{RT}=rN\left[-\frac{\tilde{\rho }}{\hat{T\;}}+\frac{\left(1-\tilde{\rho }\right)ln\left(1-\tilde{\rho }\right)}{\tilde{\rho }}+1\right]+N\tilde{\rho }\overset{t}{\underset{j=1}{\sum }}x_{j}ln\left(\frac{\varphi_{j}}{x_{j}}\right)+N\left(z-1-ln\left(z\right)\right)$$

where:

(21) $$\varphi_{j}=\frac{r_{j}N_{j}}{rN}=\frac{r_{j}x_{j}}{r}$$

represents the close-packed volumetric fraction of the *j*-th component and *n* represents the total number of moles.

We remark here that expressions (19) and (20) represent only a first step in the development of the RALF model. At the end of this section, we will discuss a further correction of the Gibbs energy expression introduced by Brandani to properly re-adapt the SL model approach to the case of an adsorbent solid phase (see Equation (30)).

In Equations (19) and (20), the reduced temperature $\tilde{T}$ and pressure $\tilde{P}$ are, respectively, given by:

(22a) $$\tilde{T}=\frac{T}{T^{*}}$$

(22b) $$\tilde{P}=\frac{P}{P^{*}}$$

Moreover, *z* is the non-equilibrium compressibility factor that, in the framework of the SL model, is given by [112,113,114]:

(23) $$z\equiv \frac{PV}{n_{t}RT}=r\frac{\tilde{P}}{\tilde{T}\tilde{\rho }}$$

where *n_t_* represents the total number of moles. If the mixture density would be allowed to attain its equilibrium value, then one would have:

(24) $$z\equiv \frac{PV}{n_{t}RT}=r\frac{\tilde{P}}{\tilde{T}\tilde{\rho }}=z^{EOS}$$

where:

(25) $$z^{EOS}-1=r\left[-\frac{\tilde{\rho }}{\tilde{T}}-\frac{ln\left(1-\tilde{\rho }\right)}{\tilde{\rho }}-1\right]+\tilde{\rho }\overset{t}{\underset{j=1}{\sum }}x_{j}ln\left(\frac{\varphi_{j}}{x_{j}}\right)$$

In the SL model, $RT^{*}$ and $P^{*}$ represent the characteristic energy and the characteristic energy density of the mixture which, in this model, are only ascribed to “mean-field” interactions. The following expression relates these two characteristic parameters:

(26) $$P^{*}v^{*}=RT^{*}$$

Such an expression holds for any multicomponent phase including the pure component. Based on Equations (13), (14) and (26), only one mixing rule concerning $T^{*}$ or, alternatively, $P^{*}$ needs to be specified. In the framework of the version of SL on which the RALF model is rooted, the following mixing rule is adopted for $P^{*}$ [109,110]:

(27) $$P^{*}=\overset{t}{\underset{j=1}{\sum }}\sum_{k=1}^{t}\varphi_{j}\varphi_{k}P_{jk}^{*}$$

where:

(28) $$P_{jk}^{*}=P_{kj}^{*}=\left(1-k_{kj}\right)\sqrt{P_{kk\;}^{*}P_{jj\;}^{*}}$$

In Equations (27) and (28), when *j* = *k*, the corresponding $P_{jj}^{*}$ represents the characteristic pressure $P_{j}^{*}$ of the component *j* and the corresponding $k_{jj}$ is equal to zero. Moreover, from Equation (28) it follows that $k_{kj}=k_{jk}$.

In conclusion, the proposed mixing rule for $P^{*}$ introduces a set of dimensionless parameters, $k_{jk}$, each one defined for a given couple of components of the multicomponent phase of interest. Therefore, for the case of *t* components, there is a set of $k_{jk}\;$parameters (with *j* ≠ *k*) in a number of $t\left(t-1\right)/2$, which represent, in general, a set of optimization parameters of the model for the system of interest along with the set formed by the (*t* − 1) $\xi_{jA}$ parameters. According to the proposed mixing rule, any$\;k_{kj}$ (with *j* ≠ *k*) can be considered as characteristic of the specific couple of components involved and, regarding the penetrant components, in the RALF model it is still assumed to be the same both in the adsorbent phase and in the external fluid phase, as it occurs in the SL model.

In addition to the correction proposed by Brandani for the characteristic parameters $v_{j}^{*}$ of the adsorbate, $P_{j}^{*}$ and $T_{j}^{*}$ should also be corrected. However, to minimize the number of fitting parameters and based on the physical meaning of the term $RT_{j}^{*}$, in the original implementation of the RALF model it has been assumed that $T_{j}^{*}$ takes the same value associated with the bulk fluid phase, where no geometrical constraints are present. Moreover, following the line of thought of the original SL model, it has been also assumed that the relationship (28) still holds in the adsorbent phase for each adsorbent. Consequently, when the correction given by Equation (18) for $v_{j}^{*}$ in the adsorbent phase is implemented, the corresponding $P_{j}^{*}$ will be consistently lower by the factor $\frac{1}{1+\xi_{jA}}$.

In conclusion, the RALF model introduces $\left(t-1\right)/\left(\frac{t}{2}+1\right)$ optimization parameters (the set of $k_{jk}\;$with *j* ≠ *k* and the set of $\xi_{jA}$). In order to adopt the model in a predictive fashion for the case of adsorption in Cu-BTC, each one of the described optimization parameters should be retrieved through a non-linear regression procedure to fit solubility data of any other system containing the couple of components associated.

We remark here that, since the RALF model has been developed for multicomponent systems, the sub-case of a pure fluid component is included in all its expressions. In particular, the expressions for *G* of multicomponent fluid mixtures, for the chemical potentials, and for the compressibility factor *z* naturally recover the case of pure component in the limit of its molar fraction approaching to 1, and all the mixing rules adopted converge consistently to the corresponding pure component value.

In the expressions reported above, each summation includes all the *t* components of the phase of interest. It is worth noting that the RALF model does not consider for any possible adsorption process occurring on the surfaces of the solid crystals. In fact, the latter contribution is expected to be, as a first approximation, negligible in comparison to the largely prevailing contribution of adsorption within the bulk phase of the solid crystals. Hence, in the case of the adsorbent phase, Brandani [109] assumes that, according to the homogeneous approach of the LF model proposed, the solid is formed by a single huge molecule (so-called ‘single bulk monocrystal’ assumption). Operatively, in using the model for data correlation purposes, the surface adsorption contribution as well as the polycrystallinity dispersion are lumped, in the form of an effective “averaged” additional contribution, with the bulk adsorption contribution. In certain cases, this approximation could result in some inconsistent values of best fitting parameters and, consequently, could determine some deviations from the experimental data when the model is used for a fully predictive approach, as it will be discussed in Section 4.

Having assumed that the solid is formed by a single molecule, the number of solid molecules *N_s_* in the continuous LF approach tends to zero. Consequently, its molecular mass diverges. Finally, since the cell volume $v_{s}^{*}$, the mixture cell volume $v^{*}$, and the close-packed pure solid volume $\;V_{S}^{*0}\;$are described by real numbers, the number of lattice sites occupied by the solid in the pure lattice ($r_{s}^{0})$ as well as in the mixtures ($r_{s}$) diverges. In fact, from Equation (9), the following relationship is derived:

(29) $$r_{s}v^{*}N_{s}=r_{s}^{0}v_{j}^{*}N_{s}=V_{S}^{*0}=\frac{m_{s}}{\rho_{s}^{*}}$$

where $\rho_{s}^{*}$ represents the close-packed density of the solid component. Equation (29) allows us to determine the limit condition for the term *r_s_N*_s_ from which, in principle, it is possible to re-express also the *rN* term in the limit condition. Alternatively, the limit expression of *rN* can be straightforwardly obtained by observing that Equation (15) can be recast as:

(30) $$\overset{t}{\underset{j=1}{\sum }}r_{j}N_{j}v^{*}=\left(\overset{t-1}{\underset{i=1}{\sum }}r_{i}N_{i}+r_{s}N_{s}\right)v^{*}=rNv^{*}=V^{*}$$

Then, from Equations (4), (7) and (30), rN can be expressed by the finite positive quantity given by:

(31) $$rN=\frac{m_{S}\;\tilde{\rho }}{\rho_{S}v^{*}}=V_{s}\frac{\;\tilde{\rho }}{v^{*}}=V\frac{\;\tilde{\rho }}{v^{*}}$$

Moreover, as indicated by Brandani, in the limit condition of $N_{s}$ →0, the SL expression of the residual Gibbs energy given by Equation (20) needs to be properly re-adapted for the adsorbent phase. In fact, in the derivation of the RALF model [109,110], *G^R,SL^* has been modified considering that, since $N_{s}\to 0$ and the solid is assumed perfectly rigid, the adsorbent component cannot contribute to the combinatorial term but has the effect of reducing the volume available to the adsorbent molecules within the lattice, as also expressed by the proposed scaling of the $v_{j}^{*}$ of the penetrants. To properly account for this issue, in the RALF model the combinatorial part of the *G^R^* is ad hoc modified with respect to the SL expression adopted for the external fluid phase (given by Equation (20)), according to the following expression [109]:

(32) $$\frac{G_{A}^{R}\left(P,T,N\right)}{RT}=rN\left[-\frac{\tilde{\rho }}{\hat{T\;}}+\frac{\left(1-\tilde{\rho }\right)ln\left(1-\tilde{\rho }\right)}{\tilde{\rho }}+1\right]+N\tilde{\rho }\overset{t-1}{\underset{i=1}{\sum }}x_{i}ln\left(\frac{\varphi_{i}}{\left(1-\varphi_{s}\right)x_{i}}\right)+N\left(z-1-ln\left(z\right)\right)$$

In Equation (32), the index *i* replaces the index *j* of Equation (20). In fact, *i* refers only to the *t* − 1 adsorbate components while the solid component is explicitly labelled with subscript *s*. It is worth noting that the expression of the compressibility factor, *z*, in the case of the adsorbent phase, is still provided by Equation (23).

Equation (32), under the hypothesis that the solid “frozen” volume is coincident with the adsorbent phase volume, represents the starting point of the RALF model to consistently derive the expression of the residual chemical potential contribution of any penetrant present in the adsorbent phase, which is the only contribution required for the calculation of the solubility isotherms. As for the penetrant chemical potential in the external fluid phase, the expressions of the residual penetrant chemical potentials are provided by performing the derivative of Equation (20). In the following, we briefly discuss the operative expressions of the penetrant chemical potential contribution required for sorption thermodynamics calculations. Regarding the external fluid phase, the expression of the equilibrium residual chemical potentials on a molecular basis for the component *k*, $\mu_{k}^{R,eq}$, can be calculated by determining the derivative of the equilibrium expression of residual *G* in an ***N***, *P*, *T* ensemble, i.e.,

(33) $$G^{R,eq}\left(T,P,N\right)\equiv G^{R}\left(T,P,N,V\left(T,P,N\right)\right)$$

where $V\left(T,P,N\right)$ is given by the EoS (Equation (24)) and $G^{R}\left(T,P,N,V\left(T,P,N\right)\right)$ is given by Equation (20). Hereafter, the superscript *eq* stands for equilibrium. The functional dependence $V\left(T,P,N\right)$ in the model under consideration is not available in a closed form. However, we recall that the equilibrium volume *V* of the mixture in a T,P,N ensemble is provided by the minimization condition of *G* towards the phase volume (i.e., the EoS) [112,113,114] and that the dependence of *G* from the mixture volume is only present in the *G^R^* term [109,131,132]. Therefore, according to the derivative chain rules, the following relationship is obtained:

(34) $$\begin{array}{cc} \frac{\mu_{k}^{R,eq}}{kT}\equiv & \frac{1}{kT}{\left(\frac{\partial G^{R,eq}}{\partial N_{k}}\right)}_{T,P,N_{j\neq k}}\\ & \;\;\;=\frac{1}{kT}{\left(\frac{\partial G^{R}}{\partial N_{k}}\right)}_{T,P,V,\;N_{j\neq k}}+\frac{1}{kT}{\left(\frac{\partial G^{R}}{\partial V}\right)}_{T,P,N}\cdot {\left(\frac{\partial V}{\partial N_{k}}\right)}_{T,P,N_{j\neq k}}\\ & \;\;\;=\frac{1}{kT}{\left(\frac{\partial G^{R}}{\partial N_{k}}\right)}_{T,P,V,\;N_{j\neq k}} \end{array}$$

In Equation (34), the expression of $V\left(T,P,N\right)$ is determined, as already discussed, by the minimization condition ${\left(\frac{\partial G^{R}}{\partial V}\right)}_{T,P,N}=0$. The last term of Equation (34) provides the operative expression of $\mu_{k}^{R,eq}$ which is obtained by coupling the following general non-equilibrium expression (which can be calculated in closed form):

(35) $$\mu_{k}^{R}={\left(\frac{\partial G^{R}}{\partial N_{k}}\right)}_{T,P,V,\;N_{j\neq k}}$$

with the EoS (Equation (24)). In particular, the following expression holds in the case of the external fluid phase:

(36a) $$\begin{array}{c} \frac{\mu_{k,F}^{R}}{kT}=-\frac{{\tilde{\rho }}_{F}}{{\tilde{T}}_{F}}r_{kF}\left(2\frac{\sum_{i=1}^{t-1}\varphi_{i}P_{ik}^{*}}{P_{F}^{*}}-1\right)+r_{k}^{0}\left[\frac{\left(1-{\tilde{\rho }}_{F}\right)ln\left(1-{\tilde{\rho }}_{F}\right)}{{\tilde{\rho }}_{F}}+1\right]\\ +\frac{r_{k}}{r_{F}}\left(r_{F}\frac{{\tilde{P}}_{F}}{{\tilde{\rho }}_{F}{\tilde{T}}_{F}}-1\right)-ln\left(z\right)+{\tilde{\rho }}_{F}\left(ln\frac{r_{k}}{r_{F}}+1-\frac{r_{k}}{r_{F}}\right) \end{array}$$

where the subscript *F* stands for fluid phase and *z* and ${\hat{\rho }}_{F}$ are obtained by solving Equations (24) and (25). In the case that the external phase is mono-component, Equation (36a) becomes:

(36b) $$\frac{\mu_{k,F}^{R}}{kT}=r_{k}^{0}\left[-\frac{{\tilde{\rho }}_{k}}{{\tilde{T}}_{k}}+\frac{\left(1-{\tilde{\rho }}_{k}\right)ln\left(1-{\tilde{\rho }}_{k}\right)}{{\tilde{\rho }}_{k}}+1\right]+z-1-ln\left(z\right)$$

Regarding the adsorbent phase, the volume of the mixture has been fixed to be in an out-of-equilibrium state and the solid is assumed insoluble in the external fluid phase. These are the same conditions hypothesized in the case of the NELF model. Consequently, Brandani [115,116,117,127] infers that in the RALF model, the phase equilibrium conditions between the external fluid phase and the adsorbate phase are dictated by the same set of equations determined for the case of the NELF model when dealing with the phase equilibrium between a multicomponent fluid phase and the glassy polymer-penetrant phase kinetically locked in an out-of-equilibrium state.

The NELF phase equilibrium conditions impose that for each adsorbate, the equilibrium chemical potentials in the external phase are equal to the non-equilibrium (NE) chemical potentials in the polymer-penetrant phase. In particular, the appropriate non-equilibrium chemical potential required in the NELF model is provided for the *k*-th penetrant by [115,116,117,127]:

(37) $$\mu_{k}^{NE}={\left(\frac{\partial G^{SL}}{\partial N_{k}}\right)}_{T,P,V,\;N_{j\neq k}}$$

where in this case, *V* is the assumed out-of-equilibrium fixed value of mixture volume, taken coincident with the out-of-equilibrium value of the pure polymer right before the sorption test.

Invoking the same approach of the NELF, the RALF model phase equilibrium condition is dictated by the following set of equations expressed in terms of residual chemical potentials [109,110]:

(38) $$\frac{\mu_{k,F}^{R,eq}}{kT}-\frac{\mu_{k}^{R}}{kT}=ln\frac{y_{k}}{x_{k}}\;\mathrm{for}\;k=1,\;2,\ldots ,\;t-1$$

where *y_k_* and *x_k_* refers to the molar fraction of the *k*-th adsorbate in the external fluid phase and in the adsorbent phase, respectively.

In Equation (38), $\mu_{k,F}^{R,eq}$ is given by Equation (36a,b) coupled with Equation (2). $\mu_{k}^{R}$ is referred to the adsorbent phase and it is still provided by Equation (35) but, differently from the equilibrium external phase case, the *G^R^* adopted for its calculation is provided by Equation (32) coupled with the non-equilibrium expression of *z* (Equation (23)). The associated value of volume to be used in these expressions is given by Equation (4).

We remark that in the case of the NELF model, the condition of phase equilibrium based upon the non-equilibrium expression of the chemical potentials provided by Equation (37) has been proven to be thermodynamically consistent with the constraints imposed by the second law of the thermodynamics on the whole biphasic system considered based on the thermodynamics with internal state variables [115,116,117]. In this respect, a well-established constitutive class for the function expressing the kinetic evolution of the volume mixture, provided by a viscoelastic model, was adopted [115,116,117]. Conversely, in the RALF approach the equivalent condition given by Equation (38) does not rely upon such kind of analysis, but it is inferred based on the same formal conditions used in the NELF model regarding the “frozen” mixture volume.

In order to close the phase equilibrium problem when using the RALF model, in the following we need to focus on the operative calculation of $\mu_{k}^{R}$. As a consequence of the discussed limit condition $N_{s}\to 0$, some comments are in order regarding the calculation of the derivative in Equation (35) in the case of the adsorbent phase. In principle, starting from Equation (32) coupled with Equations (23) and (24), the limit condition just imposes that *rN* is expressed by Equation (31) and that *N* approaches the total number of adsorbate molecules *N_p_*. In this way, the functional dependence $G^{R}\left(T,P,N,\;V\right)$ can be formally re-expressed as $G^{R}\left(T,P,N_{p},m_{s},\rho_{s},\;V\right)=G^{R}\left(T,P,N_{p},\;V\right)$, where $N_{p}$ is the subvector of N referred only to the adsorbate components, and the last identity of Equation (31) has been applied to lump$\;m_{s}\;$and $\rho_{s}$ in terms of *V*. The expression of $G^{R}\left(T,P,N_{p},m_{s},\rho_{s},\;V\right)\;$can be, therefore, equivalently adopted in the derivative of Equation (35) in order to obtain $\mu_{k}^{R}$, since neither $m_{s}$ nor $\rho_{s}$ depends on $N_{p}$. In this way, the operative expression of $\mu_{k}^{R}$ is provided by:

(39) $$\frac{\mu_{k}^{R}}{kT}=-\frac{\tilde{\rho }}{\tilde{T}}r_{k}\left(2\frac{\sum_{j=1}^{t}\varphi_{j}P_{kj}^{*}}{P^{*}}-1\right)+\left[\frac{\left(1-\tilde{\rho }\right)ln\left(1-\tilde{\rho }\right)}{\tilde{\rho }}+1\right]r_{k}^{0}+\frac{r_{k}}{r}\left(z^{EoS}-1\right)\;-ln\left(z\right)+\tilde{\rho }\left(ln\left(\frac{r_{k}}{r\left(1-\varphi_{s}\right)}\right)+1-\frac{r_{k}}{r\left(1-\varphi_{s}\right)}\right)$$

with:

(40) $$z^{EOS}-1=r\left[-\frac{\tilde{\rho }}{\tilde{T}}-\frac{ln\left(1-\tilde{\rho }\right)}{\tilde{\rho }}-1\right]+\tilde{\rho }\overset{t-1}{\underset{i=1}{\sum }}x_{i}ln\left(\frac{\varphi_{i}}{x_{i}\left(1-\varphi_{s}\right)}\right)$$

Incidentally, we observe that, in the case of a pure fluid, Equation (40) collapses into the original version of SL EoS [112,113,114]. We recall that in Equations (39) and (40), the volume *V* is assigned according to Equation (4) so that $\tilde{\rho }$ is directly provided as:

(41) $$\tilde{\rho }=\frac{\rho_{s}}{\rho^{*}\omega_{s}}=\frac{m_{s}}{\rho^{*}\omega_{s}V}$$

In Equations (39) and (40), the close-packed volumetric fraction of the solid is given by:

(42) $$\varphi_{s}\equiv \frac{r_{s}N_{s}}{r_{s}N_{s}+\sum_{i=1}^{t-1}r_{i}N_{i}}=\frac{r_{s}x_{s}}{r_{s}x_{s}+\sum_{i=1}^{t-1}r_{i}x_{i}}$$

where the limit condition $N_{s}\to 0$ imposed to obtain the last member is equivalent to divide for *N* and *N_p_*. Finally, according to Equation (29), the term $r_{s}x_{s}$ can be expressed as:

(43) $$r_{s}x_{s}=\frac{m_{s}}{Nv^{*}\rho_{s}^{*}}=\frac{m_{s}}{N_{p}v^{*}\rho_{s}^{*}}$$

where the last identity follows from the recalled limit condition. From Equation (31), in the limit condition, r can be expressed as:

(44) $$r=V\frac{\;\tilde{\rho }}{Nv^{*}}=V\frac{\;\tilde{\rho }}{N_{p}v^{*}}=\frac{m_{s}\;\tilde{\rho }}{N_{p}v^{*}\rho_{s}}$$

Equations (43) and (44) introduce the ratio $N_{p}/m_{s}$: an intensive quantity expressing the total number of molecules of adsorbate per mass of solid. This is in line with the proposed approach in which, being that the solid mass is not soluble in the external phase, the adsorbate concentration vector can be expressed per solid mass.

In the case of a single adsorbate, in Equations (39) and (40), the conditions $x_{1}=1$ and $\frac{r_{1}}{r}=\varphi_{1}=1-\varphi_{s}$ hold, so that Equations (39) and (40) result in the single equation:

(45) $$\frac{\mu_{1}^{R}}{kT}=-\frac{\tilde{\rho }}{\tilde{T}}r_{1}\left(2\frac{\sum_{j=1}^{t}\varphi_{j}P_{kj}^{*}}{P^{*}}-1\right)+\left[\frac{\left(1-\tilde{\rho }\right)ln\left(1-\tilde{\rho }\right)}{\tilde{\rho }}+1\right]r_{1}^{0}+r_{1}\left[-\frac{\tilde{\rho }}{\tilde{T}}-\frac{ln\left(1-\tilde{\rho }\right)}{\tilde{\rho }}-1\right]-ln\left(z\right)$$

### 2.2. RALFHB Model

The RALF model is based upon an LF statistics which accounts only for “mean-field” interactions between the component molecules, assuming that the empty sites do not contribute to the interaction energy. In the following, we propose an extension of the original RALF model aimed at describing the possible presence of self- and cross-specific interactions (i.e., Hydrogen-Bonding and/or Lewis acid–bases interactions) between the components, referred in the following as the RALFHB model. On the basis of the discussed formal correspondence between the NELF and the RALF models, the proposed extension follows the same procedure adopted by Mensitieri et al. [127,133] in extending the original “mean-field” version of the NELF model to the case of sorption thermodynamics of glassy polymer-penetrant mixtures displaying possible self- and cross-HB interactions. This latter extension is referred to as the NELFHB model [133]. In the following, we briefly report the fundamentals of the RALFHB approach. Full details on the procedure for the derivation of NELFHB model (and consequently, of the RALFHB one), which is rooted in thermodynamics with internal state variables, are reported in [127,133].

The first Step of the NELFHB, as well as of the RALFHB model, requires a general non-equilibrium expression of *G* accounting for both mean-field and specific interactions. This expression is provided by Panayiotou and Sanchez (PS) [134] who proposed a model based upon the factorization of the partition function between a “mean-field” contribution and an excess contribution related to specific interactions. In fact, this factorization results in two additive contributions that provide the expression for *G*: one “mean -field” LF term (*G^LF^*), which is similar to the one provided by the SL model but that can be in principle provided by any “mean-field” model, and an additional term (*G^HB^*) accounting for specific interactions, which is provided by the Veytsman [135] statistics [134,135]. Its extension to deal with sorption thermodynamics of polymer-penetrant mixtures characterized by the presence of strong interactions and kinetically locked in an out-of-equilibrium glassy state is, hereafter, referred to as the NELFHB model. A similar approach can be used to develop a model for adsorption thermodynamics in a rigid adsorbent phase where strong interactions occur. In this way, the RALFHB model is derived from the RALF approach in the same way in which the NELFHB model is obtained from the NELF approach.

Regarding the RALFHB model, consistently with the RALF model case, we have adopted for $G^{LF}$ the expression given by Equations (20) and (32), respectively, for the external fluid phase and for the adsorbent phase, taking the original *G^HB^* term of the PS model for both the phases. The expression of $G^{HB}$ can be found in the original literature of PS model [134] and reads:

(46) $$\frac{G^{HB}}{kT}=rN\left\{\overset{m}{\underset{\alpha =1}{\sum }}\overset{n}{\underset{\beta =1}{\sum }}\nu_{\alpha \beta }\left[1+\frac{G_{\alpha \beta }^{0}}{RT}+ln\left(\frac{\tilde{v}\;\nu_{\alpha \beta }}{\nu_{\alpha 0}\nu_{0\beta }}\right)\right]+\overset{m}{\underset{\alpha =1}{\sum }}\nu_{d}^{\alpha }ln\frac{\nu_{\alpha 0}}{\nu_{d}^{\alpha }}+\overset{n}{\underset{\beta =1}{\sum }}\nu_{a}^{\beta }ln\frac{\nu_{0\beta }}{\nu_{a}^{\beta }}\right\}$$

In Equation (46), $\nu_{\alpha \beta }\equiv \frac{N_{\alpha \beta }^{H}\;}{rN}$, where$\;N_{\alpha \beta }^{H}\;$represents the total number of contacts between a proton donor of kind α and a proton acceptor of kind β; $\nu_{\alpha 0}\equiv \frac{N_{\alpha 0}^{H}\;}{rN}$, where $N_{\alpha 0}^{H}\;$represents the total number of proton donors of kind α not involved in any HB interactions; $\nu_{0\beta }\equiv \frac{N_{0\beta }^{H}\;}{rN}$, where $N_{0\beta }^{H}$ represents the total number of proton acceptors of kind β not involved in any HB interactions. $\nu_{d}^{\alpha }\equiv \frac{N_{d}^{\alpha }}{rN}$, where $N_{d}^{\alpha }$ represents the total number of proton donors of kind α in the system and $\nu_{a}^{\beta }\equiv \frac{N_{a}^{\beta }}{rN}$, where $N_{a}^{\beta }$ represents the total number of proton acceptors of kind β in the system. Finally, *m* and *n* represent the total number of different typologies of proton donors and proton acceptors, respectively, present within the system.

Hereafter, $G_{\alpha \beta }^{0}$ represents the molar Gibbs energy of formation of the specific interaction between a proton donor of kind α and a proton acceptor of kind β. We remark that the energies of formation of HB must be considered as an excess energy contribution with respect to the mean-field energy which is described by the LF contribution.

Therefore, the HB contribution to G introduces in principle m×n HB formation energies, $G_{\alpha \beta }^{0}$, where each one is defined by:

(47) $$G_{\alpha \beta }^{0}=U_{\alpha \beta }^{0}+PV_{\alpha \beta }^{0}-TS_{\alpha \beta }^{0}$$

With $U_{\alpha \beta }^{0}$, $V_{\alpha \beta }^{0}$, and $S_{\alpha \beta }^{0}$, respectively, representing the molar internal energy, the molar volume, and the molar entropy of formation of the specific interaction between a proton donor of kind *α* and a proton acceptor of kind *β*. According to a well-established approach [127], the assumption $V_{\alpha \beta }^{0}=0$ is commonly adopted, so that, according to Equation (47), the HB contribution introduces $2\left(m\times n\right)$ energetic parameters, provided by the two sets of $U_{\alpha \beta }^{0}$ and $S_{\alpha \beta }^{0}$ terms. The values of such parameters regarding self- and cross-HB interactions between the adsorbates, in principle, can be obtained by regressions of VLE equilibrium data according to the version of PS model implemented for the external phase. Therefore, in an ***N**, P, T* ensemble the general non-equilibrium expression of G can be formally expressed as [133]:

(48) $$G\left(T,P,N,V,{\underline{N}}_{\alpha \beta }^{H}\right)$$

where the generic component $N_{\alpha \beta }^{H}\;$of the set of variables ${\underline{N}}_{\alpha \beta }^{H}$ represents the total number of specific interactions between a proton donor (or Lewis acid) of kind *α* and a proton acceptor (or Lewis base) of kind *β*. V is the mixture volume and ${\underline{N}}_{\alpha \beta }^{H}$ represents the set of thermodynamics internal variables of the model. The equilibrium condition of the phase of interest is dictated by the minimization of G towards the whole set of internal state variables [127,133,134]:

(49a) $${\left(\frac{\partial G}{\partial V}\right)}_{P,T,N,V,{\underline{N}}_{\alpha \beta }^{H}}=0\;\;\;\;\;\;\;(\mathrm{EoS})$$

(49b) $${\left(\frac{\partial G}{\partial N_{\alpha \beta }^{H}}\right)}_{P,T,N,\;V,\;N_{\gamma \delta \neq \alpha \beta }^{H}}=0\;\;\;\;\;\;\;\alpha =1,\;2,\ldots ,\;m\;\mathrm{and}\;\beta =1,\;2\ldots ,\;n$$

Equation (49a,b) represents a set of non-linear algebraic equations. Regarding the external fluid phase, according to the same procedure illustrated for the case of the RALF model, the residual term of the equilibrium chemical potential of the *k*-th species reads:

(50) $$\begin{array}{c} \frac{\mu_{k}^{R,eq}}{kT}\equiv \frac{1}{kT}{\left(\frac{\partial G^{R,eq}}{\partial N_{k}}\right)}_{T,P,N_{j\neq k}}=\frac{1}{kT}{\left(\frac{\partial G^{R}}{\partial N_{k}}\right)}_{T,P,V,N_{j\neq k},{\underline{N}}_{\alpha \beta }^{H}}+\frac{1}{kT}{\left(\frac{\partial G^{R}}{\partial V}\right)}_{T,P,N,{\underline{N}}_{\alpha \beta }^{H}}\cdot {\left(\frac{\partial V}{\partial N_{k}}\right)}_{T,P,N_{j\neq k}}+\\ \frac{1}{kT}\sum_{\alpha =1}^{m}\sum_{\beta =1}^{n}{\left(\frac{\partial G^{R}}{\partial N_{\alpha \beta }^{H}}\right)}_{P,T,N,V,{\underline{N}}_{\gamma \delta \neq \alpha \beta }^{H}}\cdot {\left(\frac{\partial N_{\alpha \beta }^{H}}{\partial N_{k}}\right)}_{P,T,V,N_{j\neq k}}=\frac{1}{kT}{\left(\frac{\partial G^{R}}{\partial N_{k}}\right)}_{T,P,V,N_{j\neq k},{\underline{N}}_{\alpha \beta }^{H}} \end{array}$$

where:

(51) $$G^{R,eq}\equiv G^{R}\left(N,P,T,V\left(N,P,T\right),{\underline{N}}_{\alpha \beta }^{H}\left(N,P,T\right)\right)$$

and $V\left(N,P,T\right)$ and ${\underline{N}}_{\alpha \beta }^{H}\left(N,P,T\right)$ are, in principle, provided by the solutions of Equation (49a,b). Equation (50) follows from Equation (49a,b) since the dependence of G from V and ${\underline{N}}_{\alpha \beta }^{H}$ is only involved in the calculation of the $G^{R}$ term [132,133,134].

The last member of Equation (50), which can be expressed in a closed form, coupled with Equation (49a,b), therefore, provides the operative expression of $\mu_{k}^{R,eq}$ for the equilibrium external fluid phase, thus circumventing the problem derived from the fact that the solution of Equation (49a,b) is available only numerically.

To close the sorption thermodynamics modeling, we need to focus now on the adsorbent phase. As discussed, in framework of the RALF model as well as of the NELF model, it is assumed that the adsorbent phase does not follow the equilibrium condition of the LF model adopted. In the case of the NELFHB model and, consequently, of the RALFHB model, this results in assuming that Equation (49a,b) does not hold. In principle, under the assumption of a “frozen” adsorbent phase, by following the procedure used to develop the NELFHB model in the case where the specific interactions are accounted for by the additional set of internal variables ${\underline{N}}_{\alpha \beta }^{H},\;$one needs to provide the fixed out-of-equilibrium values of V and of ${\underline{N}}_{\alpha \beta }^{H}$. Commonly, the latter information is not available so that an “instantaneous equilibrium” assumption occurs [127,133]. In fact, in the NELFHB model and, consequently, in the proposed RALFHB model, in view of the microscopic scale regarding the nature of the HB contacts that allows for a very fast rearrangement of the interactions, it is assumed that ${\underline{N}}_{\alpha \beta }^{H}$ still follows the equilibrium condition dictated by Equation (49b), but in correspondence with the fixed out-of-equilibrium mixture volume V (i.e., it is assumed that Equation (49a) does not hold) [127,133].

For the adsorbent phase, under the $N_{s}\to 0$ assumption, the general out-of-equilibrium residual Gibbs energy of the model, $G^{R}$ is now given by:

(52) $$G^{R}\equiv G^{R,LFHB}\left(N_{P},P,T,V,{\underline{N}}_{\alpha \beta }^{H}\left(N_{P}P,T,V\right)\right)=G^{R}\left(N_{P},P,T,V\right)$$

where ${\underline{N}}_{\alpha \beta }^{H}\left(N_{P},P,T,V\right)$ is, in principle, provided by the solutions of Equation (49b), V is the fixed mixture volume, and $G^{R,LFHB}(N_{P},P,T,V,{\underline{N}}_{\alpha \beta }^{H})$ is the sum of the $G^{LF}$ term given by Equation (32) and of the $G^{HB}$ term of the original PS and NELFHB models.

The expression for$\;G^{R}\left(N_{P},P,T,V\right)$ obtained under the HB instantaneous equilibrium assumption, therefore, represents the general out-of-equilibrium expression of a model which displays the volume *V* as the only internal state variable. Consequently, the problem has been traced back to the well-established NELF framework already adopted in the RALF model, and the out-of-equilibrium residual chemical potential of *k*-th adsorbate, $\mu_{k}^{R}$, is provided by derivation of $G^{R}\left(N_{P},P,T,V\right)$, according to the expression (35).

However, the solution of the sub-set (49b) in correspondence with the fixed *V* is in general available only numerically so that $G^{R}\left(N_{P},P,T,V\right)$ is not available in a closed form. To circumvent this problem, the calculation of $\mu_{k}^{R}$ can be performed as:

(53) $$\begin{array}{cc} \frac{\mu_{k}^{R}}{kT}\equiv & \frac{1}{kT}{\left(\frac{\partial G^{R}}{\partial N_{k}}\right)}_{T,P,V,\;N_{j\neq k}}\\ & \;\;\;=\frac{1}{kT}{\left(\frac{\partial G^{R,LFHB}}{\partial N_{k}}\right)}_{T,P,V,\;N_{j\neq k},{\underline{N}}_{\alpha \beta }^{H}}\\ & \;\;\;+\frac{1}{kT}\overset{m}{\underset{\alpha =1}{\sum }}\overset{n}{\underset{\beta =1}{\sum }}{\left(\frac{\partial G^{R,LFHB}}{\partial N_{\alpha \beta }^{H}}\right)}_{P,T,N_{p},\;V,N_{\gamma \delta \neq \alpha \beta }^{H}}\cdot {\left(\frac{\partial N_{\alpha \beta }^{H}}{\partial N_{k}}\right)}_{P,T,V,N_{j\neq k}}\\ & \;\;\;=\frac{1}{kT}{\left(\frac{\partial G^{R,LFHB}}{\partial N_{k}}\right)}_{T,P,V,\;N_{j\neq k},{\underline{N}}_{\alpha \beta }^{H}} \end{array}$$

that has been obtained by applying Equation (49b). Equation (53) results from the fact that the dependence of *G* upon ${\underline{N}}_{\alpha \beta }^{H}$ involves only the residual term $G^{R}$. The last term in Equation (53) can be now expressed in a closed form. Operatively, the value of $\mu_{k}^{R}$ is provided by Equation (53) coupled with Equation (49b).

Again, since the instantaneous equilibrium assumption formally brings back the formulation of the model to the original NELF framework, the set of Equation (38) properly re-casted in terms of the RALFHB model, still dictates the phase equilibrium state (i.e., the sorption thermodynamics of the adsorbates within the rigid adsorbent phase).

In the following, we show the operative expression of the residual chemical potentials. To this aim, it is worth noting that:

(54) $$G^{R}=G^{tot}-G^{id}=G^{LF}+G^{HB}-G^{id}=G^{R,LF}+G^{HB}$$

where *G^id^* represents the ideal gas term of the Gibbs energy and *G^(R,LF)^* represents the residual Gibbs energy of the mean-field LF contribution, whose expression is provided by Equations (20) and (32), respectively, in the case of the external fluid phase and the adsorbent phase. Therefore, in the calculation of the residual equilibrium chemical potentials of the *k*-th penetrant in the external fluid phase, from Equation (50) it follows:

(55) $$\mu_{k,F}^{R,eq}=\mu_{k,F}^{R,LF}+\mu_{k,F}^{R,HB}$$

where:

(56) $$\mu_{k,F}^{R,LF}\equiv {\left(\frac{\partial G^{R,LF}}{\partial N_{k}}\right)}_{T,P,V,\;N_{j\neq k},{\underline{N}}_{\alpha \beta }^{H}}={\left(\frac{\partial G^{R,LF}}{\partial N_{k}}\right)}_{T,P,V,\;N_{j\neq k}}$$

and

(57) $$\mu_{k,F}^{R,HB}\equiv {\left(\frac{\partial G^{R,HB}}{\partial N_{k}}\right)}_{T,P,V,\;N_{j\neq k},{\underline{N}}_{\alpha \beta }^{H}}$$

Equations (56) and (57) need to be coupled with Equation (49a,b) and the subscript *F* has been added to underline that this is referred to as the fluid phase. 

Analogously, for the adsorbent phase, from Equation (53) it follows that:

(58) $$\mu_{k}^{R}=\mu_{k}^{R,LF}+\mu_{k}^{R,HB}$$

where:

(59) $$\mu_{k}^{R,LF}={\left(\frac{\partial G^{R,LF}}{\partial N_{k}}\right)}_{T,P,V,\;N_{j\neq k},{\underline{N}}_{\alpha \beta }^{H}}={\left(\frac{\partial G^{R,LF}}{\partial N_{k}}\right)}_{T,P,V,\;N_{j\neq k}}$$

and

(60) $$\mu_{k}^{R,HB}={\left(\frac{\partial G^{R,HB}}{\partial N_{k}}\right)}_{T,P,V,\;N_{j\neq k},{\underline{N}}_{\alpha \beta }^{H}}$$

As before, Equations (59) and (60) need to be coupled with Equation (49b) with the assigned volume *V* provided by Equation (4). It is worth noting that the last identities in Equations (56) and (59) follow from the fact that the general non-equilibrium expressions of $G^{LF}$ in each phase do not depend upon ${\underline{N}}_{\alpha \beta }^{H}$ [127,133,134]. Consequently, the formal expressions of $\mu_{k,F}^{R,LF}$ and of $\mu_{k}^{R,LF}$ coincide with the corresponding ones of the RALF model reported in the previous section, i.e., Equations (36a) and (39) for the fluid phase and the adsorbent phase, respectively. In the following, we report the operative expressions of $\mu_{k,F}^{R,HB}$ and $\mu_{k}^{R,HB}$ along with the minimization conditions represented by Equation (49) which are required to close the phase equilibrium problem. We observe that Equations (57) and (59) are formally given by the same expression. Hence, by following the same procedure of refs. [133,134], one obtains:

(61) $$\frac{\mu_{k,F}^{R,HB}}{kT}=\frac{\mu_{k}^{R,HB}}{kT}=r_{k}^{0}\nu_{H}+\overset{m}{\underset{\alpha =1}{\sum }}d_{a}^{k}ln\frac{\nu_{\alpha o}}{\nu_{d}^{\alpha }}+\overset{n}{\underset{\beta =1}{\sum }}a_{\beta }^{k}ln\frac{\nu_{0\beta }}{\nu_{a}^{\beta }}$$

where $d_{a}^{k}$ and $a_{\beta }^{k}\;$represent, respectively, the number of donors of kind *α* and of acceptors of kind *β* present on a molecule of species *k*-th, and we have also that:

(62) $$\nu_{H}=\overset{m}{\underset{\alpha =1}{\sum }}\overset{n}{\underset{\beta =1}{\sum }}\nu_{\alpha \beta }$$

The minimization conditions represented by Equation (49a) imply that:

(63) $$\tilde{P}+{\tilde{\rho }}^{2}+\;\tilde{T}\;\left[ln\left(1-\tilde{\rho }\right)+\tilde{\rho }\left(1-\frac{1}{r}+\nu_{H}\right)\right]-\frac{\tilde{T}{\tilde{\rho }}^{2}}{r}\overset{t}{\underset{j=1}{\sum }}x_{j}ln\frac{\varphi_{j}}{x_{j}}=0$$

where, in obtaining the HB contribution it can be followed by the procedure reported in [127,133].

In the case of a pure fluid, Equation (63) collapses into the equation of state of the PS model [134]. Finally, Equation (49b), according to [127,133], implies that:

(64) $$\frac{\tilde{v}\;\nu_{\alpha \beta }}{\nu_{\alpha 0}\;\nu_{0\beta }}=exp\left(-\frac{G_{\alpha \beta }^{H}}{RT}\right)\;\mathrm{for}\;\mathrm{each}\;\alpha =1,\;2,\ldots ,\;m\;\mathrm{and}\;\beta =1,\;2\ldots ,\;n$$

On the basis of the previous discussion, for the external fluid phase, Equation (61) is intended to be coupled with Equations (63) and (64), and, for the adsorbent phase, Equation (61) is intended to be coupled only with Equation (64) while the fixed volume V is provided by Equation (4).

We conclude this section addressing an issue which concerns the application of both the RALF model and its extension, the RALFHB model. In fact, from inspection of the shape of the expressions of the adsorbate chemical potentials in the adsorbent phase (i.e., Equation (39) in the case of the RALF model and Equation (61) and Equation (39) coupled with Equation (64) in the case of the RALFHB model), it is evident that the simultaneous dependence on the out-of-equilibrium volume, *V* = *V_S_*, and on the mass of solid, *m_s_*, can be expressed as a dependence only on the solid mass density, *ρ_S_*. This result is expected since the chemical potentials are intensive properties. Operatively, the following relationship can be used:

(65) $$\rho_{S}={\left(\frac{1}{\rho_{s}^{*}}+V_{m}\right)}^{-1}$$

In Equation (65), $\rho_{s}^{*}$ and $V_{m}\;$represent, in the framework of the RALF approach, the solid skeletal density and the pore volume of the solid per mass of solid itself, respectively. Both the adsorbent parameters can be measured experimentally, as it will be discussed in Section 4. Finally, we observe that the dependence on $\rho_{s}\;$is, in turn, only expressed as a dependence on the $\tilde{\rho }$ of the adsorbent phase mixture, which can be simply calculated as:

(66) $$\tilde{\rho }=\frac{\rho_{s}}{\rho^{*}\omega_{s}}$$

### 2.3. Dual-Site Extension of the RALF Approach: The RALF-DS and RALFHB-DS Models

In this section, we deal with the extension of the original RALF approach to the case of a heterogeneous rigid adsorbent displaying different kinds of adsorbent cages. Indeed, as detailed in the Introduction section, this is the case of the Cu-BTC MOF.

Firstly, we briefly examine the extension of the RALF approach proposed by Brandani [136] to address the case of a dual cage system, which results in the RALF dual solid model (RALF-DS). As it will be evident in the following, this approach can be easily extended to any multi-cage case once the required information on the heterogenous structure of the rigid adsorbent as well of the corresponding set of model parameters are available.

The fundamental assumption is that the two kinds of adsorbent cages contribute in parallel to the adsorption process. In the DS approach, these two different families of cages are ascribed to two different “fictive” solids. The whole adsorption isotherm of the actual rigid adsorbent for a given external fluid phase is then calculated as the averaged sum of the contributions associated to each fictive solid. To this regard, each fictive solid can be, in turn, modeled by using the RALF model or the RALFHB model in the case that specific interactions are expected. Following the approach of Brandani [136] to develop the RALF-DS model, we propose here the RALFHB-DS model in which the behavior of each fictive solid is described using the RALFHB model.

The implementation of the RALF-DS model introduces two additional parameters as compared to the homogeneous RALF model, namely, the mass fraction of fictive solid *I* with respect to the total solid mass of the actual solid (indicated as $\omega_{I})$ and the fraction of pore volume of fictive solid *I* with respect to the total pore volume of the actual solid (indicated as $f_{vI})$. The density of each fictive solid is given by [136]:

(67) $$\rho_{S}={\left(\frac{1}{\rho_{s}^{*}}+\frac{f_{V}V_{m}}{\omega }\right)}^{-1}$$

In Equation (67), $\frac{1}{\rho_{s}^{*}}$ represents the close-packed specific volume of the fictive solid of interest while $\frac{f_{V}V_{m}}{\omega }$ provides the volume of voids of the fictive solid of interest per mass of the whole solid. $V_{m}$ represents the total pore volume of the actual solid per total mass of the whole solid. Indeed, this parameter can be estimated by experimental investigations or, alternatively, it represents a further fitting parameter of the DS model.

Once Equation (67) has provided the value of the $\rho_{S}$ for each fictive solid, one can implement the RALF model or the RALFHB model to calculate the related sorption isotherm expressed per mass of the fictive solid of interest $m_{s}$. For a given *k*-th adsorbate this can be expressed for any fictive solid as $m_{k}/m_{s}$. Finally, by using the parameter ω the overall solubility of the *k*-th adsorbate in the actual solid is given by:

(68) $$\frac{m_{k,tot}}{m_{s,tot}}=\frac{\omega_{I}m_{k,I}}{m_{s,I}}+\frac{(1-\omega_{I})m_{k,II}}{m_{s,II}}$$

where $m_{k,tot}$ refers to the total mass of adsorbate *k*-th in the actual solid and $m_{s,tot}$ is the total mass of the actual solid. Finally, the subscripts *I* and *II* refer to fictive solids *I* and *II,* respectively.

### 2.4. Summary of the Theoretical Approaches

Empirical and semiempirical modeling have been proposed in the literature to describe sorption in porous materials in general, and in MOFs in particular. These approaches provide a quick and effortless evaluation of the adsorption phenomena. Relevant examples are the Langmuir–Freundlich (LF) isotherm, characterized however by a thermodynamic inconsistency in the low-pressure region, the Virial–Langmuir (VL) model which allows us to gather some information regarding the nature of the adsorbate–adsorbent interaction, and the Dubinin–Astakhov (DA) model which provides a fair prediction of multicomponent isotherms

Semiempirical thermodynamics models, despite their good fitting capability, suffer from a lack of thermodynamic consistency in dealing with adsorbates molecules with an appreciable difference in size and do not account in a full predictive fashion for the adsorbate–adsorbate and/or adsorbate–adsorbent interactions. Moreover, they are not suitable for a fully predictive approach since their adjustable parameters are not rooted in a rigorous physical background.

To provide a robust background on the mechanisms taking place during the gas adsorption on MOF materials and an interpretation of their relative vibrational spectra, over the last decade different ab initio computational methods have been implemented which allowed for the identification of the adsorption sites. For Cu-BTC frameworks, many contributions have been produced in this direction using DFT, GCMC, and MD calculations, but a large computational effort is required for these calculations with MOFs.

To overcome this drawback, a lattice fluid equation of state theory firmly rooted on a statistical thermodynamics background aimed at modeling the adsorption thermodynamics of multicomponent fluid mixtures within a rigid adsorbent has been recently proposed in the literature. This approach, known as RALF, has been successfully applied to mixtures of gases and/or vapors within rigid zeolites and MOF systems. The RALF model is an ad hoc extension of the original SL multicomponent Lattice Fluid model but, differently from SL theory, in the RALF approach, the volume of the adsorbent–adsorbate system is not provided by an EoS and is assumed to be constant. In view of its structure, the RALF model is intrinsically a pure mean-field theory. Then, based the on RALF approach, we propose here an extension of this model to deal with cases where specific adsorbates–adsorbates and adsorbates–adsorbent interactions need to be accounted for, the RALFHB model.

Finally, to deal with the case of heterogeneity of adsorption sites, we have shown how one can easily extend both the RALF and the RALFHB models by considering different kinds of adsorbent cages and introducing the so-called ‘Dual-Solid’ or ‘Multi-Solid’ models.

## 3. Experimental Section

In this section, we summarize the information on the material and procedures we adopted to obtain in our lab, some experimental data that are discussed in Section 4, and the literature data.

### 3.1. Materials

Basolite^®^ C300 [Copper benzene-1,3,5-tricarboxylate in powder form was provided by Sigma-Aldrich (Milano, Italy). According to the supplier, the specific surface area was in the range of 1500–2100 m^2^/g, the bulk density was 0.35 g/cm^−3^, and the average particle size of the crystalline powder was 15.96 μm. Carbon dioxide with 99.99% purity was supplied by SOL S.p.A. (Monza, Italy). Chloroform with 99.8% purity and Potassium Bromide (KBr) windows 2.0 mm thick with a diameter of 13 mm were supplied by Sigma-Aldrich (Milano, Italy).

A Cu-BTC dispersion was prepared from a 5.0 wt% mixture of Cu-BTC in chloroform stirred for 2 h and further sonicated for 30 min. Samples for spectroscopy measurements were prepared by casting a few drops of the Cu-BTC dispersion on a KBr window allowing solvent evaporation for 2 h at room temperature. Afterwards, the window was slightly pressed at 5 bar for compacting the MOF layer, thus optimizing the FTIR transmission measurement.

### 3.2. FTIR Spectroscopy

The FTIR measurements were performed under gas flowing using a modified Linkam cell, THMS350V (Surrey, UK), equipped with temperature control (83–623 K) and a vacuum system. The cell was connected through service lines to a mass-flow-controller [MKS Type GM50A (Andover, MA, USA)] to set the CO_2_ flux, while a solenoid valve regulated the downstream pressure. The system was equipped with a Pirani vacuometer and an MKS Baratron 121 pressure transducer (full scale 1000 Torr, resolution 0.01 Torr, accuracy ±0.5% of the reading) (Andover, MA, USA). Further details on the experimental apparatus are reported in [137]. The equipment allowed for the in situ activation of the sample at 150 °C under a vacuum and the collection of isothermal data at different temperatures. The diffusion cell was coupled to a Spectrum-100 FTIR spectrometer (Perkin-Elmer, Norwalk, CT, USA), equipped with a beam splitter made of a thin Ge film supported on KBr plates and a wide-band DTGS detector.

## 4. Results and Discussion

### 4.1. Analysis of Cu-BTC/CO_2_ Systems by FTIR Spectroscopy

**Figure 2:**
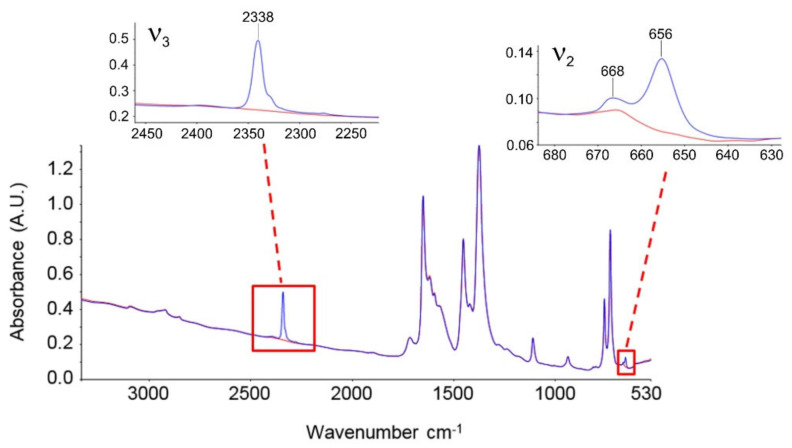
Absorbance spectra of activated CuBTC (red trace) and CuBTC equilibrated at 150 Torr, 35 °C. Insets display the ν_3_ and ν_2_ bands of sorbed CO_2_.

A comparison between the spectra of Cu-BTC after activation (red trace, see Figure 2) and after equilibration at 35 °C with an external CO_2_ pressure of 150 Torr (blue trace, see Figure 2) highlights the signals produced by the guest molecule at 2338 cm^−1^ (O=C=O antisymmetric stretching, ν_3_) and at 656 cm^−1^ (O=C=O bending, ν_2_).

The absorbance of the ν_3_ band is linearly dependent on the amount of sorbed CO_2_, as demonstrated by correlating the sorption isotherm monitored spectroscopically with that from an independent volumetric measurement (see Figure S1, Supplementary Materials [137]). This correlation allows for the calibration of the photometric observable according to the Beer–Lambert relationship and validates the quantitative analysis by spectroscopic means.

The ν_3_ bandshape provides further information on the molecular environment. A pronounced shoulder is detected at about 2324 cm^−1^; previous studies [138,139] demonstrated that this feature is a (ν_3_ + ν_2_) − ν_2_ hot-band enhanced by Fermi resonance with the nearby fundamental. It does not originate from a CO_2_ population distinct from that producing the main signal and, after suitable resolution, can be neglected in the bandshape analysis. The main component at 2338 cm^−1^ displays a lower resolution and a full-width-at-half-height (FWHH) considerably larger than those observed in various host/guest systems of the same kind (see, for instance, a comparison with the polyetherimide/CO_2_ system represented in Figure S2, Supplementary Materials [140]). This effect is indicative of a multicomponent bandshape with an unresolved fine structure. Resolution-enhancement approaches are needed to analyze such complex profiles: we adopted two-dimensional correlation spectroscopy (2D-COS), a well-established tool very effective in diffusion studies for investigating weakly interacting systems [141,142,143]. The resolution enhancement brought about by 2D-COS originates from the spreading of the spectral data over a second frequency axis, coupled with the vanishing of the asynchronous correlation intensity for signals evolving at the same rate. In addition, the technique provides information on the evolution of the system as a function of the perturbing variable (CO_2_ concentration, in the present case) [144].

**Figure 3:**
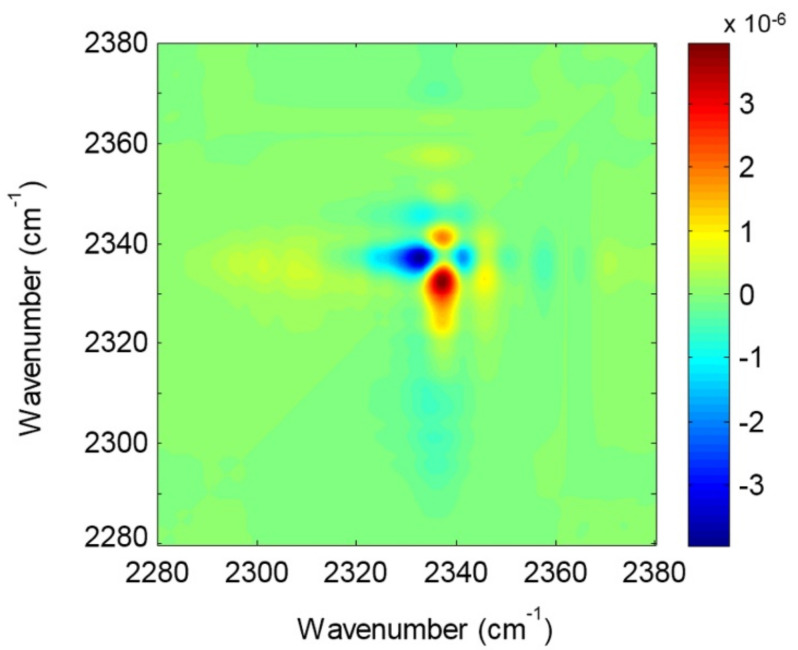
Asynchronous correlation spectrum in the 2280–2380 cm^−1^ range relative to the isothermal measurement at 0 °C.

The asynchronous map relative to isothermal measurements at 0 °C is represented in Figure 3. It displays a well-resolved four-peak pattern that reveals the presence of three components at 2337, 2346, and 2333 cm^−1^. These correspond to probe populations that interact with different chemical environments in the nanostructure; the diverse interactions promote the separation of the ν_3_ signal in three components and induce a difference in terms of molecular mobility of the guest molecules that grants the detection by 2D-COS. This conclusion has been confirmed by the analysis of the bending mode in the 670–640 cm^−1^ range [137] and has been associated with the occurrence of three specific adsorption sites, theoretically predicted by first-principles studies, that are characterized by distinct values of the interaction energy [144].

The FTIR analysis also reveals that the CO_2_/HKUST-1 interactions are weak. In fact, the perturbation induced to the ν_3_ mode is unable to produce a sizable resolution of the components, not even in the form of shoulders or bandshape asymmetry. The occurrence of multiple components can only be evidenced by applying 2D-COS spectroscopy. Furthermore, the ν_3_ peak position is close to that observed in weakly interacting systems (see Figure S2, Supplementary Materials).

### 4.2. Modeling Sorption Isotherms of Low Molecular Weight Compounds in Cu-BTC

In this section several experimental results available in the literature for sorption isotherms of gases and vapors in Cu-BTC and their interpretation with RALF, RALF-DS, RALFHB, and RALFHH-DS are presented and reviewed.

To the aim of modeling the adsorption of CO_2_ and CH_3_OH in Cu-BTC by using the RALF approach, one needs to provide structural and energetic parameters of the model. In particular, the RALF model requires pure LF parameters of the adsorbent solid: the close-packed density ($\rho_{s}^{*}$), $T_{s}^{*}$, and $P_{s}^{*}$. Since this set of parameters cannot be found through the fitting of dilatometric equilibrium data of the solid, the common procedure consists of estimating the pure solid parameters through the simultaneous non-linear regression of solubility data of several penetrants [137]. According to this procedure, in the present contribution we have directly implemented the model to fit simultaneously the solubility of light weakly interacting gases (O_2_, CH_4_, N_2_, and CO_2_) and specific interacting vapor (CH_3_OH).

Based on the Cu-BTC structure and the kinetic diameters of the adsorbates considered (reported in Table 3), three different cages are, in principle, involved in the adsorption process of the investigated light gases and vapor.

In the case of weakly interacting gases, the observed type I adsorption behavior [147] suggests that the penetrants independently explore and access the different cages of the framework under observation and are not affected by the presence of the different guest–host interactions related to the double Cu^2+^ paddle-wheel. Consequently, a ‘single solid’ RALF model can be applied to describe the mean-field adsorption process within the whole solid. Conversely, in the case of CH_3_OH, according to [148], specific interactions are expected to occur between Cu^2+^ and hydroxyl group of methanol. Due to heterogenous spatial distribution of the copper doublet (L2 with respect to L3 and S1), the methanol molecules probe different energetic environments when exploring the polar cages (L2) and the apolar cages (L3 and S1) [58]; indeed, this is confirmed by the observed type IV adsorption behavior [147]. To account for this heterogenous solid behavior, in the case of methanol, the ‘dual solid’ approach has been implemented, combining the RALFHB model, used for describing the polar cages contribution, with the pure mean-field RALF model, used for describing the apolar cages contribution. Cross- and self-specific interactions have been modeled by assuming that each hydroxyl group of methanol has 1 proton donor $\left(d_{1}^{1}\right)$ and 1 proton acceptor $\left(a_{1}^{1}\right)$; each copper of Cu-BTC has 2 proton donors $\left(d_{2}^{S}\right)$. For the sake of comparison, we have also implemented, in the case of methanol, the RALF-DS model in which both solids have been modeled disregarding HB contribution.

The set of optimization parameters adopted in the simultaneous fitting procedure is formed by: $T_{s}^{*}$, $P_{s}^{*}$, $k_{CO_{2}-S},$ $k_{CH_{4}-S}$, $k_{N_{2}-S}$, $k_{O_{2}-S}$, $k_{CH_{3}OH-S}^{I}$, $k_{CH_{3}OH-S}^{II}$, $\zeta_{CO_{2}-S},$ $\zeta_{CH_{4}-S}$, $\zeta_{N_{2}-S}$, $\zeta_{O_{2}-S}$, $\zeta_{CH_{3}OH-S}^{I}$, $\zeta_{CH_{3}OH-S}^{II}$, $U_{CH_{3}OH-S}^{0}$, $S_{CH_{3}OH-S}^{0}$, $\omega_{I}$, and $f_{V,I}$. The corresponding optimized values of these parameters are reported in Table 4. We remark that, to reduce the number of fitting parameters, a single value of $T_{s}^{*}$, $P_{s}^{*}$, and $\rho_{s}^{*}$ have been adopted for both the ‘single solid’ and the ‘two fictive solids’ (I and II) models. Therefore, the whole fitting procedure is coupled for all investigated adsorbates. The energetic heterogeneity of the system in the case of methanol is taken into account for both the specific interaction contribution (present only in the solid I) and different mean-field interaction parameters of the ‘two fictive solids’ ($k_{CH_{3}OH-S}^{I}$ and $k_{CH_{3}OH-S}^{II}$). In addition, the cage’s heterogeneity, which is significant in the case of methanol, is accounted for through the two volume correction parameters ($\zeta_{CH_{3}OH-S}^{I}$ and $\zeta_{CH_{3}OH-S}^{II}$) as well as through the intrinsic double solid parameters $\omega_{I}$ and $f_{V,I}$.

In order to close the modeling, one needs to provide the skeletal solid density which in the RALF model framework corresponds to $\rho_{s}^{*}$, the pore volume, $V_{m}$, of the whole MOF expressed as pore volume per mass of the whole solid, and the RALF lattice fluid characteristic parameters, $T^{*}$, $P^{*}$, and $\rho^{*}$, of each penetrant which actually correspond to the Sanchez–Lacombe parameters, as discussed in Section 2.1. In addition, the self-specific interaction parameters $U_{CH_{3}OH-CH_{3}OH}^{0}$ and $S_{CH_{3}OH-CH_{3}OH}^{0}$ for methanol are also required. The list of the adopted values of these parameters is reported in Table 3. Regarding the methanol, two sets of parameters are reported in Table 5: the first one [112] refers to a pure mean-field RALF model and, consequently, the characteristic parameters coincide with the Sanchez–Lacombe ones, while the second set is associated with the RALFHB model and it has been retrieved in the present work by a non-linear regression of liquid–vapor equilibrium data.

**Figure 4:**
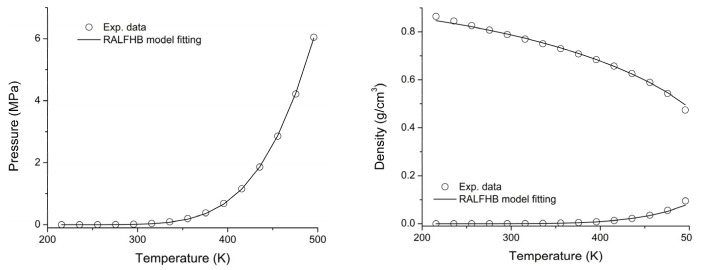
Liquid–vapor equilibrium of methanol: comparison between experimental data [149] and RALFHB model fitting.

Figure 4 shows the comparison between the experimental data for vapor pressure of methanol as a function of temperature and of density of methanol at liquid–vapor equilibrium taken from [149] and the model fitting using the optimized values of fitting parameters of the RALFHB model. It is evident that the model exhibits an excellent fitting capability in the whole range of the temperature investigated. In fact, the RALFHB model reproduces simultaneously the saturation densities of liquid and vapor phase along with the saturation pressure, provided that the data are sufficiently far away from the critical transition. We remark that the only fitting parameters are provided by the characteristic lattice fluid parameters, in fact the hydrogen-bonding formation parameters have been fixed according to the literature of PS [134]. To this regard, as discussed in Section 2.2, the RALFHB parameters for the external fluid phase coincide with the ones of the PS model in the case of the single penetrant system.

The last required input parameters for the modeling are $\rho_{s}^{*}$ and $\rho_{s}$. The first one is identified with the skeletal density of the solid and, according to its crystalline structure, is taken in the literature to be equal to 2.685 g cm^−3^ [150]; the second parameter, $\rho_{s}$*,* usually is not provided directly by the experiments but it can be calculated in the case of a single solid by Equation (65) while, in the case of the RALF-DS model, is provided by Equation (67) for each fictive solid. To this regard, the parameter $V_{m}$ (usually determined experimentally by pycnometry) is required to close the problem. $V_{m}$ depends on the way the Cu-BTC sample is prepared. For the sets of data regarding the weakly interacting gases, $V_{m}$ is equal to 0.79 cm^3^ g^−1^ [48] and for Cu-BTC/CH_3_OH sets of data, $V_{m}$ is equal to 0.731 cm^3^ g^−1^ [58].

**Figure 5:**
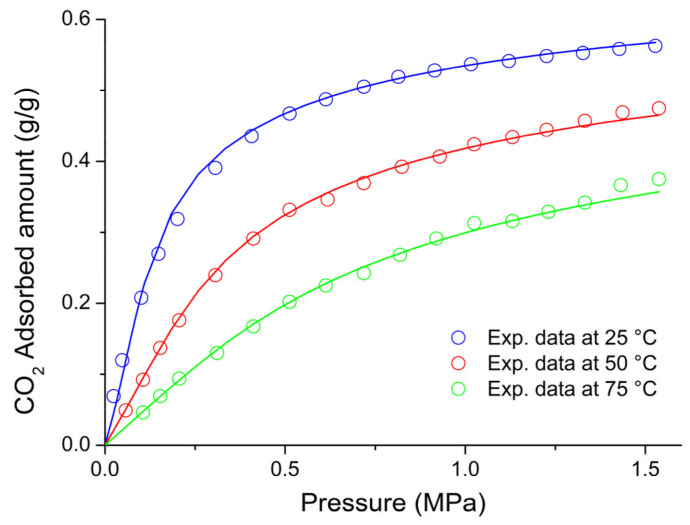
Comparison between experimental CO_2_ adsorption data in Cu-BTC [48] and simultaneous optimized RALF model fitting (solid lines).

**Figure 6:**
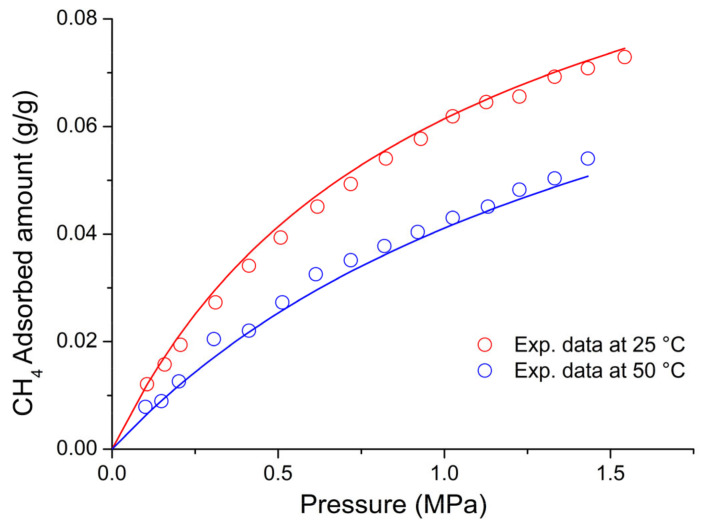
Comparison between experimental CH_4_ adsorption data in Cu-BTC [48] and simultaneous optimized RALF model fitting (solid lines).

**Figure 7:**
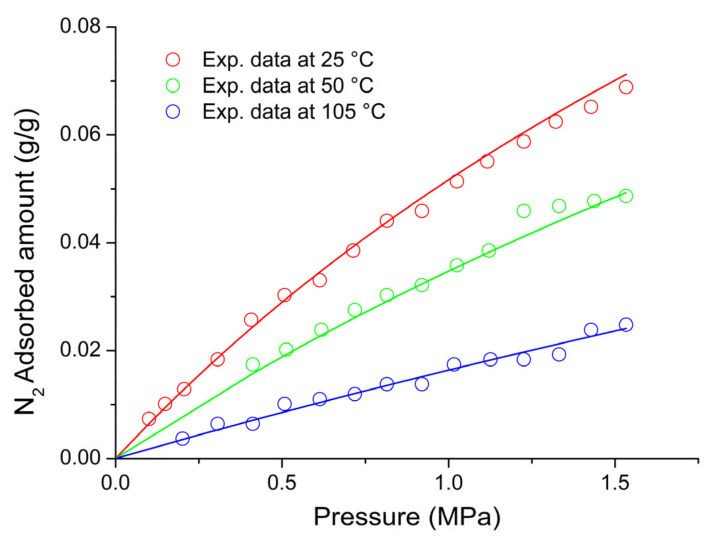
Comparison between experimental N_2_ adsorption data in Cu-BTC [48] and simultaneous optimized RALF model fitting (solid lines).

**Figure 8:**
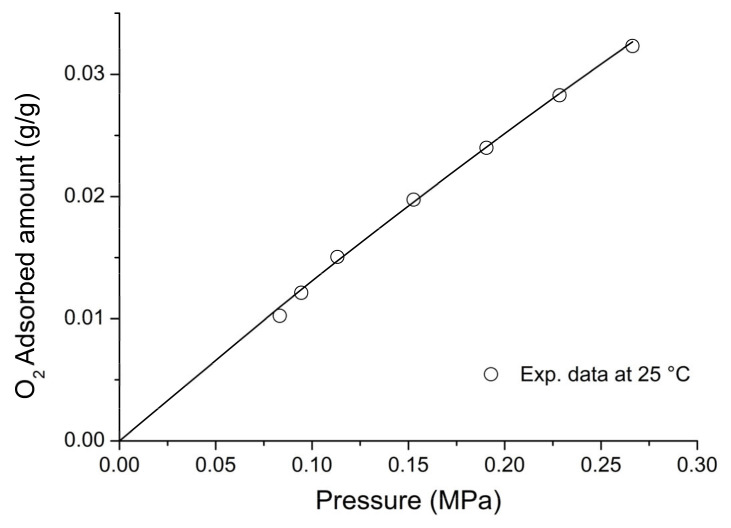
Comparison between experimental O_2_ adsorption data in Cu-BTC [48] and simultaneous optimized RALF model fitting (solid lines).

**Figure 9:**
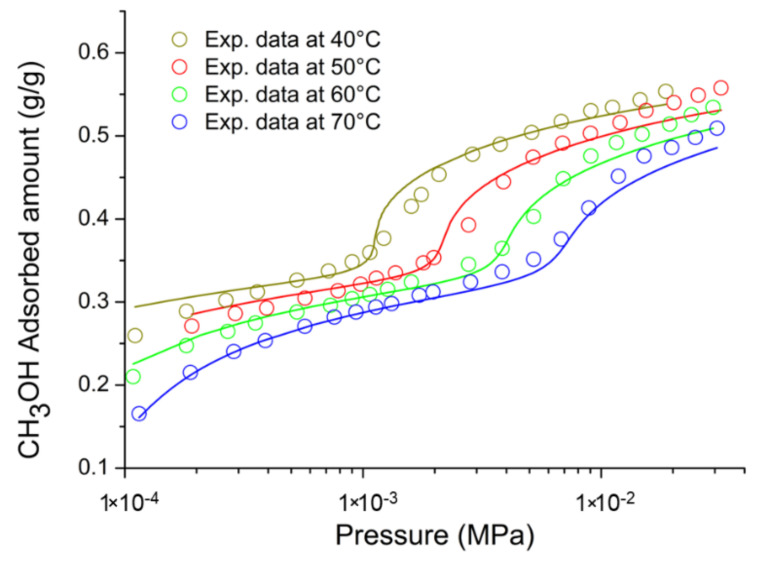
Comparison between experimental CH_3_OH adsorption data in Cu-BTC [58] and simultaneous optimized (RALF/RALFHB)-DS model fitting (solid lines).

Once all the input parameters have been provided, simultaneous fitting of all the sets of adsorption data considered can be performed. In Figure 5, Figure 6, Figure 7, Figure 8 and Figure 9, a comparison between the experimental adsorption data for the weakly interacting gases and the optimized predictions provided by the simultaneous fitting procedure is reported.

**Figure 10:**
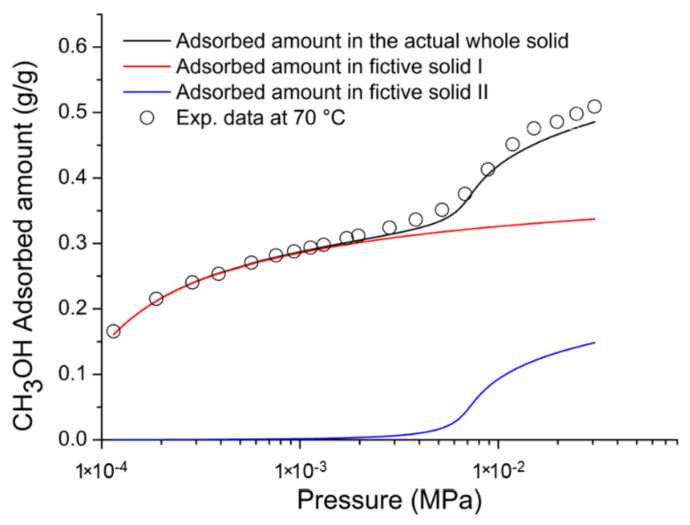
Comparison between experimental data [58] and simultaneous optimized model fitting (solid lines), showing the two additional contributions associated with the fictive solids I (RALFHB) and II (RALF).

It is evident that the modeling approach exhibits an excellent fitting capability both in the case of weakly interacting gases (in particular, reproducing well the type I behavior) and in the case of specific interacting Cu-BTC/CH_3_OH systems (in particular, reproducing well the stepwise type IV behavior commonly observed in a semi-log scale). To better elucidate the DS approach, in Figure 10, the two additive contributions associated to the fictive solids I and II at 70 °C (similar results, omitted for brevity, have been found also at the other investigated temperatures) are explicitly reported. It is worth noting that the polar solid I dominates the adsorption process at low pressures approaching a saturation plateau. This is expected in view of the strong cross-specific interactions occurring between the hydroxyl group of methanol and Cu^2+^, whose total number is limited by the stoichiometry of the MOF. Once the solid I contribution approaches its saturation limit, the stepwise contribution of the apolar solid II becomes significant allowing the overall solubility curve of type IV of the whole solid to reproduce.

It is worth noting that the optimized value of $S_{CH_{3}OH-S}^{0}$ is significantly low in comparison to the values commonly observed for hydrogen-bonding-specific interactions (see for instance the value of $S_{CH_{3}OH-CH_{3}OH}^{0}$ in Table 3). Indeed, this result is in line with the observation that the interaction between OH^−^ of methanol and Cu^2+^ is not associated to an angular constraint of the rotational mobility, differently from the case of a classic hydrogen-bonding. In the framework of Veystman [26,27] statistics, the correlated reduction in rotational mobility is associated with the entropy of formation of the specific interaction. Then, in the case of non-directional-specific interactions, as in the case of interest here, the value of the entropy of formation of the interaction is still expected to be negative but negligible. In conclusion, the values of $U_{CH_{3}OH-S}^{0}$ and $S_{CH_{3}OH-S}^{0}$ point to a quite athermal Gibbs energy of formation of cross-specific interactions, which is more negative as compared with the common values of hydrogen-bonding Gibbs energy of formation (as reported in the specific table in ref. [127]). Indeed, this is reasonable in Lewis’ acid–base involving ions and polar groups.

To better understand RALFHB model results, one can analyze the self- and cross-specific interactions occurring in the system. For instance, regarding the isotherm reported in Figure 10, at 70 °C for the solid associated with the polar cage (L2) at the lowest pressure investigated (1.148·10^−4^ MPa), the model predicts that around 77.5% of the adsorbent-specific interacting sites are involved in the cross interaction with the methanol hydroxyl, while at the highest pressure investigated (0.02486 MPa), this value raises to around 91.2%. This result is consistent with the shape of the adsorption contribution of solid I which represents the polar cages (see Figure 10). Regarding the ratio between the methanol self- and cross-specific interactions in the solid I, its value exhibits a crossover in the range of pressures investigated spanning from 0.47 to 1.49 MPa; this result is in line with the observed trend of the saturation value of the MOF-specific interacting sites and, in addition, it highlights that more non-cross interacting methanol molecules are located in the polar cages as the external pressure is increased. The modeling approach also predicts that the same qualitative trend of the methanol self-specific interacting molecules as a function of the pressure is observed in the solid II, which represents the apolar cages. Similar results are observed at the other temperatures investigated and, in particular, by reducing the temperature, an increase in both self- and cross-specific interactions of the methanol in each solid occurs and this effect is more prominent for the interactions characterized by the lower entropy of formation. In fact, in the case of cross-specific interactions the behavior is quite athermal.

**Figure 11:**
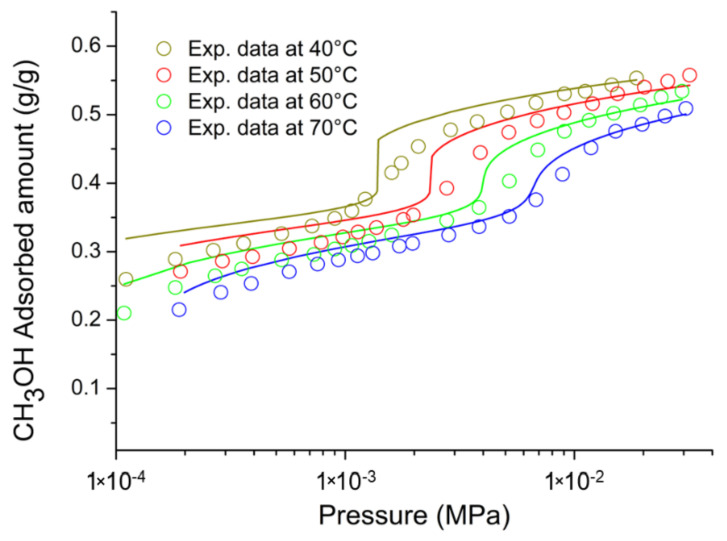
Comparison between experimental data [58] and simultaneous optimized model fitting (solid lines).

For the sake of comparison, we have also implemented a simultaneous fitting procedure of the whole set of data disregarding the contribution of specific interactions, i.e., by applying the pure mean-field RALF model also in describing the polar cage in the case of the Cu-BTC/CH_3_OH system, but still retaining the DS approach. Figure 11 reports the comparison between the experimental data and the optimized fitting results for the Cu-BTC/CH_3_OH systems. It is evident that disregarding the HB contribution does not allow us to properly reproduce quantitatively the experimental data, particularly at low pressure, where the contribution of Cu-BTC-CH_3_OH cross-HB interaction is expected to be significant.

Regarding the weakly interacting gases, the correlated predictions are quite like the ones reported for the previous simultaneous fitting case (i.e., the ones reported in Figure 5, Figure 6, Figure 7 and Figure 8). For the sake of brevity, the table reporting the optimized parameters for the simultaneous fitting performed with the pure mean-field RALF model and the figures showing the comparison of the experimental data of the weakly interacting gases with the optimized prediction curves are reported in the Supplementary Materials (see Figures S3–S7 and Table S1).

To further test the robustness of the RALF type of approach, based on the values of the optimized fitting parameters reported in Table 4, we have implemented the model in a fully predictive fashion to reproduce five CO_2_ adsorption isotherms of two Cu-BTC systems which differ from the ones adopted in the previous simultaneous fitting procedure. These two Cu-BTC structures are characterized by different specific volume porosities, $V_{m}$, still retaining the same $\rho_{s}^{*}$ implemented in the case of sets of data of the fitting procedure, so that $\rho_{s}$ assumes different values.

For the CO_2_ adsorption isotherms at 10 °C, 20 °C, 45 °C, and 70 °C, $V_{m}$ is equal to 0.57 g cm^−3^ [50] and $\rho_{s}$ is calculated according to Equation (65). In the case of the CO_2_ adsorption isotherms at 35 °C, a commercial Cu-BTC (Basolite^®^ C300) which displays $\rho_{s}=1.26{\;g\;\mathrm{cm}}^{-3}$ [56] has been used.

**Figure 12:**
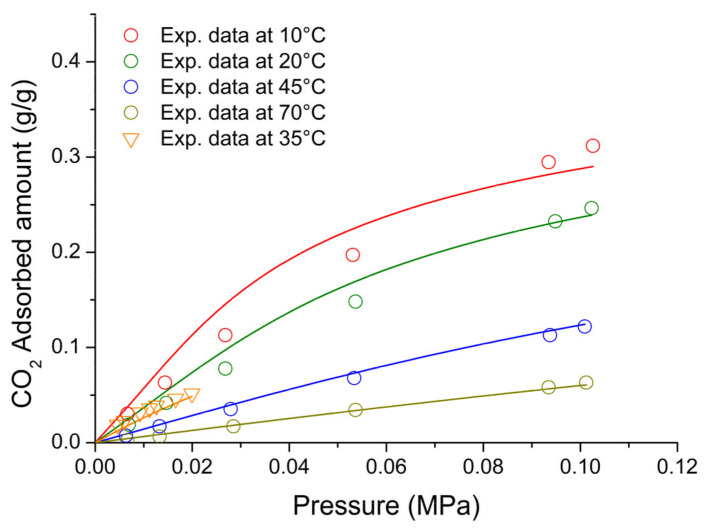
Comparison between RALF model prediction (solid lines) and experimental data of CO_2_ adsorption isotherms at 10 °C, 20 °C, 45 °C, 70 °C (taken from [50]), and 35 °C (taken from [137]).

Figure 12 proves the excellent agreement of RALF model predictions (implemented with the single solid framework) with the experimental data of CO_2_ adsorption isotherms at 10 °C, 20 °C, 45 °C, 70 °C (data taken from [50]), and 35 °C (data taken from [137]). In conclusion, by properly accounting for the actual specific porosity of the different Cu-BTC samples, the model can consistently predict the adsorption isotherms in a wide range of temperatures and pressures.

**Figure 13:**
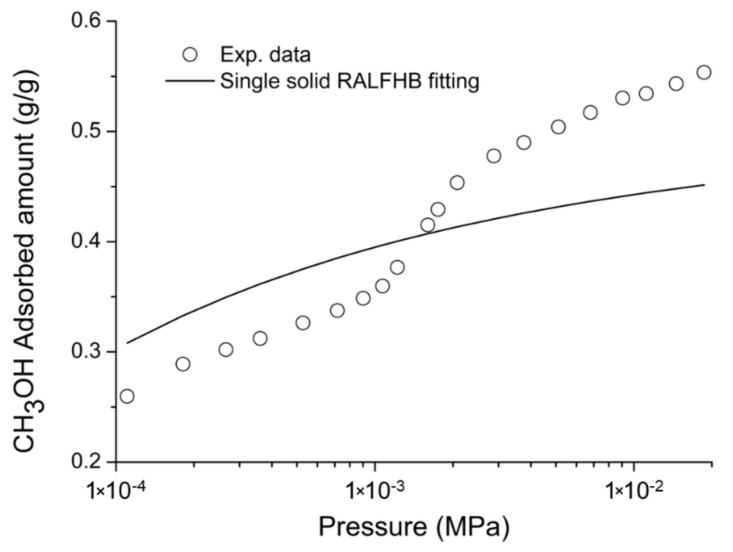
Comparison between experimental adsorption isotherm at 40 °C [58] and optimized model fitting with ‘single solid’ RALFHB model (solid line).

To better elucidate the model performance regarding the type IV behavior exhibited by the methanol isotherms, we have also investigated the fitting capability of the RALFHB model assuming a simple ‘single solid’ framework. To this aim, we have performed a simultaneous non-linear regression of the adsorption isotherms at 40 °C, 50 °C, 60 °C, and 70 °C. As an example, in Figure 13 the results of the best fitting procedure of the experimental data at 40 °C are reported.

It is evident that accounting for specific interactions in the ‘single solid’ approach is not enough to reproduce the type IV behavior, indicating that the combined energetic and structural heterogeneity of the adsorbent material should be properly considered using a multi-solid approach.

## 5. Conclusions

An overview of experimental adsorption data of low molecular weight compounds in Cu-BTC is provided, also analyzing some semiempirical and theoretical models used to interpret adsorption thermodynamics.

The theoretical approach based on the original RALF approach has been described in detail and extended to account for the contribution of specific self- and cross-interactions by introducing the new RALFHB model. This general framework allows us to account also for the heterogeneity of adsorption sites of the adsorbent materials by properly modifying the structure of the RALF and RALFHB models considering a ‘multi solid’ structure of the adsorbent material. The case of adsorption of weakly interacting gases in Cu-BTC can be dealt with simply by using the RALF theory while the thermodynamics of adsorption of methanol can be properly addressed only by considering a dual solid (DS) structure, using the RALF-DS model to account for sites heterogeneity or, better, using the RALF/RALFHB-DS model to account for both sites heterogeneity and the occurrence of specific interactions for polar sites. In this way, a theoretical ‘platform’ is made available based on the original RALF framework, well-suited for the interpretation of adsorption thermodynamics in MOFs and, in particular, in Cu-BTC.

In fact, this theoretical approach has been proven to be very effective both in fitting the adsorption data of some gases and of methanol vapor in a specific Cu-BTC sample and in providing excellent predictions for adsorption in other Cu-BTC specimens displaying a different specific volume porosity.

## Figures and Tables

**Table 1:** Morphological features of the adsorbent materials and operating conditions of the adsorbates of interest retrieved from the literature.

Adsorbent			Isotherm			
Material	Surface Area [m^2^/g]	Pore Volume [cm^3^/g]	Adsorbate	T ^a^ [K]	P ^b^ [MPa]	Refs.
**Synthesized Cu-BTC**	964.5	0.658	CO_2_, CH_4_, N_2_, O_2_, N_2_O	295	0–0.1	[12]
			H_2_O		0–3 × 10^−9^	
**Synthesized Cu-BTC**	/	0.400	H_2_	77, 87	0–0.1	[38]
			Ar	87		
**Synthesized Cu-BTC**	1239	0.620	H_2_	77, 87	0–0.1	[39]
**Synthesized Cu-BTC**	872	0.270	CO	183	1.33 × 10^−7^–1.33 × 10^−4^	[40]
			H_2_	15, 77	1 × 10^−5^–0.092	
**Synthesized Cu-BTC**	1507	0.750	H_2_	77	0–0.1	[41]
**Synthesized Cu-BTC**	/	0.684	CO_2_	150–300	0–9 × 10^−9^	[23]
			CO	60–300	0–2 × 10^−8^	
			N_2_	60–180	0–4 × 10^−8^	
			H_2_	20–150	5 × 10^−8^	
**Synthesized Cu-BTC**	/	0.72	H_2_	77	0–1	[3]
			NO	196, 298	0–0.1	
**Synthesized Cu-BTC**	>2000	/	CO_2_, CH_4_	303, 323, 373	0–0.4	[42]
**/**	/	/	CO, CH_4_, N_2_, H_2_	298	0–0.5	[43]
**Synthesized Cu-BTC**	1800	0.684	H_2_	20–300	5 × 10^−8^	[44]
**Synthesized Cu-BTC**	857, 1482	0.425, 0.753	CO_2_	295, 318	0–0.6	[45]
			N_2_, O_2_, Ar		0–1	
**Synthesized Cu-BTC**	1540	0.800	CO_2_	313, 333, 353	0.0002–0.133	[46]
**Commercial Cu-BTC**	1366	0.550	CH_4_, C_2_H_6_, N_2_, O_2_	295	0–0.1	[47]
**Synthesized Cu-BTC**	1571	0.790	CO_2_, CH_4_, N_2_	298, 323, 348, 378	0–2	[48]
			O_2_	298	0–0.3	
			H_2_O	298, 305	0–1 ^c^	
**Synthesized Cu-BTC**	1492	/	CO_2_	298	0–0.5	[49]
**Synthesized Cu-BTC**	1400	0.350	CO_2_	283, 293, 318, 343	0–0.12	[50]
**Synthesized Cu-BTC**	/	0720	CD_4_	77, 87	0–0.1	[35]
**Synthesized Cu-BTC**	/	/	CO_2_, CH_4_, N_2_	298	0–2.5	[51]
**Synthesized Cu-BTC**	2211	0.813	CO_2_, CO, CH_4_,	303	0–5	[52]
**Commercial Cu-BTC**	1270	0.710	CO_2_	308, 313, 328, 343	0–50	[53]
			CH_3_OH	298	0–0.1	
			CH_4_, N_2_, H_2_	303, 318, 333	0–50	
**Synthesized Cu-BTC**	1663	0.750	CO	295, 318, 353	0–8	[54]
			CO_2_		0–5	
			CH_4_		0–10	
**Commercial Cu-BTC**	1755	0.700	H_2_O	298, 313	0–1 ^c^	[55]
**Synthesized Cu-BTC**	/	/	CO_2_, CH_4_, N_2_	282, 295, 312, 332	0–0.6	[56]
**Commercial Cu-BTC**	1560	/	CH_3_COCH_3_	303, 313, 323	0–0.08 ^c^	[57]
**Synthesized Cu-BTC**	/	/	CH_3_OH, CH_3_(CH_2_)_4_CH_3_	313	0–0.1	[30]
**Commercial Cu-BTC**	1458	0.656	CH_3_OH, C_2_H_5_OH, CH_3_(CH_2_)_2_OH, (CH_3_)_2_CHOH, CH_3_(CH_2_)_3_OH, CH_3_(CH_2_)_5_OH, H_2_O, (CH_3_)_2_CO, CH_3_CN, (CH_2_)_4_O, (CH_3_)_2_NCH, CH_3_(CH_2_)_4_CH_3_, CH_3_(CH_2_)_5_CH_3_, C_6_H_4_(CH_3_)_2_, C_6_H_12_	323	0–0.1	[58]
**Synthesized Cu-BTC**	1073–1338	0.456–0.554	CO_2_	273, 298	0.0035–0.132	[59]
**Synthesized Cu-BTC**	1382	0.570	CO_2_, CH_4_	273	0–0.12	[60]
**Synthesized Cu-BTC**	892	0.379	CO_2_	277, 298, 318	0–1.4	[61]
**Synthesized Cu-BTC**	892	0.379	CO_2_	273, 295	0–0.1	[62]
**Synthesized Cu-BTC**	1554	/	CO_2_, CH_4_	303	0–4	[63]
**Synthesized Cu-BTC**	620	0.770	CH_3_OH, CH_3_CH_2_OH, H_2_O, CCl_4_	303	0–1 ^c^	[64]
**Cu-BTC/synthesis procedure**	/	/	O_2_, N_2_	273, 283, 298	0–0.2 ^c^	[65]
**Synthesized Cu-BTC**	1540	0.710	CH_3_OH	298	0–0.014	[66]
**Synthesized Cu-BTC**	1540	0.710	CH_3_OH, CH_3_COCH_3_	298	0–0.01	[67]
**Commercial Cu-BTC**	1522	/	CO_2_	298	0–1 ^c^	[68]
**Synthesized Cu-BTC**	/	/	CO_2_	308	0–1.2	[69]
**Synthesized Cu-BTC**	1415	0.610	CO_2_	273, 298	0–0.1	[70]
**Synthesized Cu-BTC**	1202	0.530	CO_2_	273, 298	0–0.1	[71]
**Synthesized Cu-BTC**	1180	0.530	CO_2_, CH_4_, N_2_	298, 323, 348	0–1	[72]

^a^ adsorption temperature. ^b^ adsorption pressure range. ^c^ values specified as P/P_0_.

**Table 2:** Combined experimental and theoretical approaches used to characterize the interaction between the adsorbent and adsorbate retrieved from the literature.

Method	Experimental	Theoretical Modeling/Simulation	Ref.
Piezometric sorption	Adsorption isotherms	/	[12]
SAPA	Adsorption isotherms	/	[38]
GSA	Adsorption isotherms	/	[39]
SAPA	XANES spectra, IR spectra/ Adsorption isotherms	/	[40]
GSA	Adsorption isotherms	Virial–Langmuir	[41]
FTIR	IR spectra	/	[23]
FTIR/GSA	IR spectra/ Adsorption isotherms	/	[3]
MSB	Adsorption isotherms	Langmuir	[42]
/	/	Grand Canonical Monte Carlo	[43]
FTIR	IR spectra	/	[44]
/	/	Grand Canonical Monte Carlo	[93]
MSB	Adsorption isotherms	Virial–Langmuir	[45]
GSA	Adsorption isotherms	Grand Canonical Monte Carlo	[46]
ASAP	Adsorption isotherms	Grand Canonical Monte Carlo	[47]
GSA	Adsorption isotherms	/	[48]
GSA	Adsorption isotherms	Density Functional Theory/ Grand Canonical Monte Carlo	[49]
SAPA	Adsorption isotherms	Langmuir–Freundlich	[50]
GSA	Adsorption isotherms	Grand Canonical Monte Carlo	[35]
MSB	Adsorption isotherms	Langmuir–Freundlich	[52]
GSA	Adsorption isotherms	Grand Canonical Monte Carlo	[51]
MSB	Adsorption isotherms	/	[53]
MSB	Adsorption isotherms	Virial–Langmuir	[54]
FTIR	Adsorption isotherms	/	[55]
FTIR/GSA	IR spectra/ Adsorption isotherms	Langmuir	[56]
GSA	Adsorption isotherms	Density Functional Theory	[57]
GSA	Adsorption isotherms	Langmuir–Freundlich	[30]
GSA	Adsorption isotherms	Langmuir–Freundlich/Grand Canonical Monte Carlo	[58]
SAPA	Adsorption isotherms	/	[59]
SAPA	Adsorption isotherms	Dual-site Langmuir–Freundlich	[60]
GSA	Adsorption isotherms	Toth	[61]
GSA	Adsorption isotherms	Density Functional Theory/ Grand Canonical Monte Carlo	[65]
FTIR/SAPA	IR spectra/ Adsorption isotherms	Virial–Langmuir	[62]
SAPA	Adsorption isotherms	Dubinin–Astakhov	[63]
GSA	Adsorption isotherms	Dubinin–Astakhov	[64]
/	/	Three-site Langmuir–Freundlich/Configurational-Bias Monte Carlo	[94]
GSA	Adsorption isotherms	Grand Canonical Monte Carlo	[66]
GSA	Adsorption isotherms	Dual-site Langmuir–Freundlich//Grand Canonical Monte Carlo	[67]
SAPA	Adsorption isotherms	/	[68]
GSA	Adsorption isotherms	Grand Canonical Monte Carlo	[69]
/	/	Grand Canonical Monte Carlo	[70]
SAPA	Adsorption isotherms	/	[71]
GSA	Adsorption isotherms	/	[72]

MSB = Magnetic Suspension Balance; GSA = Gravimetric Sorption Analyzer; SAPA = Surface Area and Porosity Analyzer.

**Table 3:** Kinetic diameters of the penetrants.

Compound	Kinetic Diameter [nm]	Ref.
CO_2_	0.33	[145]
CH_4_	0.38	[145]
N_2_	0.364	[145]
O_2_	0.346	[145]
CH_3_OH	0.36	[146]

**Table 4:** Optimized values of fitting parameters of RALFHB model.

$T_{s}^{*}$ [K]	1388
$P_{s}^{*}$ [MPa]	4092
$k_{CO_{2}-S}$	0.3774
$k_{CH_{4}-S}$	0.3052
$k_{N_{2}-S}$	0.2749
$k_{O_{2}-S}$	0.2115
$k_{CH_{3}OH-S}^{I}$	−0.1516
$k_{CH_{3}OH-S}^{II}$	0.2890
$\zeta_{CO_{2}-S}$	0.2606
$\zeta_{CH_{4}-S}$	0.5798
$\zeta_{N_{2}-S}$	0.2693
$\zeta_{O_{2}-S}$	0.6829
$\zeta_{CH_{3}OH-S}^{I}$	0.0318
$\zeta_{CH_{3}OH-S}^{II}$	0.0057
$U_{CH_{3}OH-S}^{0}$ [J mol^−1^]	−22036
$S_{CH_{3}OH-S}^{0}$ [J mol^−1^ K^−1^]	−0.0044
$\omega_{I}$	0.4337
$f_{V,I}$	0.6052

**Table 5:** List of input parameters.

Compound	$T^{*}$ [K]	$P^{*}$ [MPa]	$\rho^{*}$ [g cm^−3^]	$U_{CH_{3}OH-CH_{3}OH}^{0}$ [J mol^−1^]	$S_{CH_{3}OH-CH_{3}OH}^{0}$ [J mol^−1^ K^−1^]	Ref.
CO_2_	300	630	1.515	/	/	[112]
CH_4_	215	250	0.5	/	/	[112]
N_2_	145	160	0.943	/	/	[112]
O_2_	214	180	1.250	/	/	[112]
CH_3_OH (no HB)	468	1202	0.922	/	/	[112]
CH_3_OH	493	449	0.890	−25,100 ^†^	−26.5 ^†^	This work

^†^ Values are taken from [134].

## Data Availability

Data are available upon request to the corresponding author.

## Supplementary Materials

The following supporting information can be downloaded at: https://www.mdpi.com/article/10.3390/ijms23169406/s1.

## References

[1] Tran U.P., Le K.K., Phan N.T. (2011). Expanding applications of metal−organic frameworks: Zeolite imidazolate framework ZIF-8 as an efficient heterogeneous catalyst for the knoevenagel reaction. ACS Catal..

[2] Safaei M., Foroughi M.M., Ebrahimpoor N., Jahani S., Omidi A., Khatami M. (2019). A review on metal-organic frameworks: Synthesis and applications. TRAC-Trend Anal. Chem..

[3] Connolly B.M., Madden D.G., Wheatley A.E.H., Fairen-Jimenez D. (2020). Shaping the Future of Fuel: Monolithic Metal–Organic Frameworks for High-Density Gas Storage. J. Am. Chem. Soc..

[4] Cui Y., Yue Y., Qian G., Chen B. (2012). Luminescent functional metal–organic frameworks. Chem. Rev..

[5] Kreno L.E., Leong K., Farha O.K., Allendorf M., Van Duyne R.P., Hupp J.T. (2012). Metal–organic framework materials as chemical sensors. Chem. Rev..

[6] Britt D., Tranchemontagne D., Yaghi O.M. (2008). Metal-organic frameworks with high capacity and selectivity for harmful gases. Proc. Natl. Acad. Sci. USA.

[7] Peterson G.W., Wagner G.W., Balboa A., Mahle J., Sewell T., Karwacki C.J. (2009). Ammonia vapor removal by Cu_3_(BTC)_2_ and its characterization by MAS NMR. J. Phys. Chem. C..

[8] Küsgens P., Rose M., Senkovska I., Fröde H., Henschel A., Siegle S., Kaskel S. (2009). Characterization of metal-organic frameworks by water adsorption. Microporous Mesoporous Mater..

[9] Glover T.G., Peterson G.W., Schindler B.J., Britt D., Yaghi O. (2011). MOF-74 building unit has a direct impact on toxic gas adsorption. Chem. Eng. Sci..

[10] Rosen M.A., Koohi-Fayegh S. (2016). The prospects for hydrogen as an energy carrier: An overview of hydrogen energy and hydrogen energy systems. Energy Ecol. Environ..

[11] Ritchie H., Roser M., Rosado P. (2020). CO₂ and greenhouse gas emissions. Our World in Data.

[12] Wang Q.M., Shen D.M., Bulow M., Lau M.L., Deng S.G., Fitch F.R., Lemcoff N.O., Semanscin J. (2002). Metallo-Organic Molecular Sieve for Gas Separation and Purification. Microporous Mesoporous Mater..

[13] Millward A.R., Yaghi O.M. (2005). Metal–Organic Frameworks with Exceptionally High Capacity for Storage of Carbon Dioxide at Room Temperature. J. Am. Chem. Soc..

[14] Salehi S., Anbia M. (2017). High CO_2_ Adsorption Capacity and CO_2_/CH_4_ Selectivity by Nanocomposites of MOF-199. Energy Fuels.

[15] Cavenati S., Grande C.A., Rodrigues A.E. (2006). Removal of Carbon Dioxide from Natural Gas by Vacuum Pressure Swing Adsorpt. Energy Fuels.

[16] Dietzel P.D.C., Besikiotis V., Blom R. (2009). Application of Metal–Organic Frameworks with Coordinatively Unsaturated Metal Sites in Storage and Separation of Methane and Carbon Dioxide. J. Mater. Chem..

[17] Rowsell J.L.C., Yaghi O.M. (2004). Metal–organic frameworks: A new class of porous materials. Microporous Mesoporous Mater..

[18] Rosseinsky M.J. (2004). Recent developments in metal–organic framework chemistry: Design, discovery, permanent porosity and flexibility. Microporous Mesoporous Mater..

[19] Drenchev N., Ivanova E., Mihaylov M., Hadjiivanov K. (2010). CO as an IR probe molecule for characterization of copper ions in a basolite C300 MOF sample. Phys. Chem. Chem. Phys..

[20] Greathouse J.A., Allendorf M.D. (2006). The interaction of water with MOF-5 simulated by molecular dynamics. J. Am. Chem. Soc..

[21] Li J.-R., Kuppler R.J., Zhou H.-C. (2009). Selective gas adsorption and separation in metal–organic frameworks. Chem. Soc. Rev..

[22] Chui S.S.-Y., Lo S.M.-F., Charmant J.P.H., Orpen A.G., Williams I.D. (1999). A Chemically Functionalizable Nanoporous Material [Cu_3_(TMA)_2_(H_2_O)_3_]*n*. Science.

[23] Bordiga S., Regli L., Bonino F., Groppo E., Lamberti C., Xiao B., Wheatley P.S., Morris R.E., Zecchina A. (2007). Adsorption Properties of HKUST-1 Toward Hydrogen and Other Small Molecules Monitored by IR. Phys. Chem. Chem. Phys..

[24] Hu Z., Zhao D. (2017). Metal–organic frameworks with Lewis acidity: Synthesis, characterization, and catalytic applications. CrystEngComm.

[25] Yang Q., Xue C., Zhong C., Chen J.-F. (2007). Molecular simulation of separation of CO_2_ from flue gases in CU-BTC metal-organic framework. AIChE J..

[26] Siperstein F.R., Myers A.L. (2001). Mixed-gas adsorption. AIChE J..

[27] Shekhah O., Liu J., Fischer R., Wöll C. (2011). MOF thin films: Existing and future applications. Chem. Soc. Rev..

[28] Dhakshinamoorthy A., Alvaro M., Corma A., Garcia H. (2011). Delineating similarities and dissimilarities in the use of metal organic frameworks and zeolites as heterogeneous catalysts for organic reactions. Dalton Trans..

[29] Dhakshinamoorthy A., Alvaro M., Garcia H. (2012). Commercial metal–organic frameworks as heterogeneous catalysts. Chem. Commun..

[30] Van Assche T.R., Duerinck T., Gutierrez Sevillano J.J., Calero S., Baron G.V., Denayer J.F. (2013). High adsorption capacities and two-step adsorption of polar adsorbates on copper–benzene-1,3,5-tricarboxylate metal–organic framework. J. Phys. Chem. C.

[31] Chmelik C., Kärger J., Wiebcke M., Caro J., van Baten J.M., Krishna R. (2009). Adsorption and Diffusion of Alkanes in CuBTC Crystals Investigated Using Infra-red Microscopy and Molecular Simulations. Microporous Mesoporous Mater..

[32] Liu J., Wang Y., Benin A.I., Jakubczak P., Willis R.R., LeVan M.D. (2010). CO_2_/H_2_O Adsorption Equilibrium and Rates on Metal-Organic Frameworks: HKUST-1 and Ni/DOBDC. Langmuir.

[33] Wehring M., Gascon J., Dubbeldam D., Kapteijn F., Snurr R.Q., Stallmach F. (2010). Self-Diffusion Studies in CuBTC by PFG NMR and MD Simulations. J. Phys. Chem. C.

[34] Yang L., Naruke H., Yamase T. (2003). A Novel Organic/Inorganic Hybrid Nanoporous Material Incorporating Keggin-Type Polyoxometalates. Inorg. Chem. Commun..

[35] Getzschmann J., Senkovska I., Wallacher D., Tovar M., Fairen-Jimenez D., Düren T., van Baten J.M., Krishna R., Kaskel S. (2010). Methane Storage Mechanism in the Metal-Organic Framework Cu_3_(btc)_2_: An in situ Neutron Diffraction Study. Microporous Mesoporous Mater..

[36] Calero S., Gutiérrez-Sevillano J.J., García-Pérez E. (2013). Effect of the Molecular Interactions on the Separation of Nonpolar Mixtures Using Cu-BTC Metal-Organic Framework. Microporous Mesoporous Mater..

[37] Huang J., Seager S., Petkowski J.J., Zhan Z., Ranjan S. (2022). Methanol—A Poor Biosignature Gas in Exoplanet Atmospheres. Astrophys. J..

[38] Lee J., Li J., Jagiello J. (2005). Gas sorption properties of microporous metal organic frameworks. J. Solid State Chem..

[39] Krawiec P., Kramer M., Sabo M., Kunschke R., Froede H., Kaskel S. (2006). Improved Hydrogen Storage in the Metal-Organic Framework Cu_3_(BTC)_2_. Adv. Eng. Mater..

[40] Prestipino C., Regli L., Vitillo J.G., Bonino F., Damin A., Lamberti C., Zecchina A., Solari P.L., Kongshaug K.O., Bordiga S. (2006). Local structure of framework Cu (II) in HKUST-1 metallorganic framework: Spectroscopic characterization upon activation and interaction with adsorbates. Chem. Mater..

[41] Rowsell J.L., Yaghi O.M. (2006). Effects of functionalization, catenation, and variation of the metal oxide and organic linking units on the low-pressure hydrogen adsorption properties of metal−organic frameworks. J. Am. Chem. Soc..

[42] Cavenati S., Grande C.A., Rodrigues A.E., Kiener C., Müller U. (2008). Metal organic framework adsorbent for biogas upgrading. Ind. Eng. Chem. Res..

[43] Karra J.R., Walton K.S. (2008). Effect of open metal sites on adsorption of polar and nonpolar molecules in metal−organic framework Cu-BTC. Langmuir.

[44] Vitillo J.G., Regli L., Chavan S., Ricchiardi G., Spoto G., Dietzel P.D., Bordiga S., Zecchina A. (2008). Role of exposed metal sites in hydrogen storage in MOFs. J. Am. Chem. Soc..

[45] Chowdhury P., Bikkina C., Meister D., Dreisbach F., Gumma S. (2009). Comparison of adsorption isotherms on Cu-BTC metal organic frameworks synthesized from different routes. Microporous Mesoporous Mater..

[46] Farrusseng D., Daniel C., Gaudillere C., Ravon U., Schuurman Y., Mirodatos C., Dubbeldam D., Frost H., Snurr R.Q. (2009). Heats of adsorption for seven gases in three metal−organic frameworks: Systematic comparison of experiment and simulation. Langmuir.

[47] García-Pérez E., Gascón J., Morales-Flórez V., Castillo J.M., Kapteijn F., Calero S. (2009). Identification of adsorption sites in Cu-BTC by experimentation and molecular simulation. Langmuir.

[48] Liang Z., Marshall M., Chaffee A.L. (2009). CO_2_ adsorption-based separation by metal organic framework (Cu-BTC) versus zeolite (13X). Energy Fuels.

[49] Yazaydın A.O., Benin A.I., Faheem S.A., Jakubczak P., Low J.J., Willis R.R., Snurr R.Q. (2009). Enhanced CO_2_ adsorption in metal-organic frameworks via occupation of open-metal sites by coordinated water molecules. Chem. Mater..

[50] Aprea P., Caputo D., Gargiulo N., Iucolano F., Pepe F. (2010). Modeling carbon dioxide adsorption on microporous substrates: Comparison between Cu-BTC metal−organic framework and 13X zeolitic molecular sieve. J. Chem. Eng. Data.

[51] Karra J.R., Walton K.S. (2010). Molecular simulations and experimental studies of CO_2_, CO, and N_2_ adsorption in metal−organic frameworks. J. Phys. Chem. C.

[52] Hamon L., Jolimaître E., Pirngruber G.D. (2010). CO_2_ and CH_4_ separation by adsorption using Cu-BTC metal−organic framework. Ind. Eng. Chem. Res..

[53] Möllmer J., Möller A., Dreisbach F., Gläser R., Staudt R. (2011). High pressure adsorption of hydrogen, nitrogen, carbon dioxide and methane on the metal–organic framework HKUST-1. Microporous Mesoporous Mater..

[54] Chowdhury P., Mekala S., Dreisbach F., Gumma S. (2012). Adsorption of CO, CO_2_ and CH_4_ on Cu-BTC and MIL-101 metal organic frameworks: Effect of open metal sites and adsorbate polarity. Microporous Mesoporous Mater..

[55] DeCoste J.B., Peterson G.W., Schindler B.J., Killops K.L., Browe M.A., Mahle J.J. (2013). The effect of water adsorption on the structure of the carboxylate containing metal–organic frameworks Cu-BTC, Mg-MOF-74, and UiO-66. J. Mater. Chem. A.

[56] Nobar S.N., Farooq S. (2012). Experimental and modeling study of adsorption and diffusion of gases in Cu-BTC. Chem. Eng. Sci..

[57] Terencio T., Di Renzo F., Berthomieu D., Trens P. (2013). Adsorption of acetone vapor by Cu-BTC: An experimental and computational study. J. Phys. Chem. C.

[58] Van Assche T.R., Denayer J.F. (2013). Fabrication and separation performance evaluation of a metal–organic framework based microseparator device. Chem. Eng. Sci..

[59] Yang Y., Shukla P., Wang S., Rudolph V., Chen X.M., Zhu Z. (2013). Significant improvement of surface area and CO_2_ adsorption of Cu–BTC via solvent exchange activation. RSC Adv..

[60] Huang W., Zhou X., Xia Q., Peng J., Wang H., Li Z. (2014). Preparation and adsorption performance of GrO@ Cu-BTC for separation of CO_2_/CH_4_. Ind. Eng. Chem. Res..

[61] Policicchio A., Zhao Y., Zhong Q., Agostino R.G., Bandosz T.J. (2014). Cu-BTC/aminated graphite oxide composites as high-efficiency CO_2_ capture media. ACS Appl. Mater. Interfaces.

[62] Zhao Y., Seredych M., Jagiello J., Zhong Q., Bandosz T.J. (2014). Insight into the mechanism of CO_2_ adsorption on Cu–BTC and its composites with graphite oxide or aminated graphite oxide. Chem. Eng. J..

[63] Gomez L.F., Zacharia R., Bénard P., Chahine R. (2015). Multicomponent adsorption of biogas compositions containing CO_2_, CH_4_ and N_2_ on Maxsorb and Cu-BTC using extended Langmuir and Doong–Yang models. Adsorption.

[64] Kondo A., Hall A.S., Mallouk T.E., Maeda K. (2015). A New Synthetic Route to Microporous Silica with Well-Defined Pores by Replication of a Metal–Organic Framework. Chem. Eur. J..

[65] Sava Gallis D.F., Parkes M.V., Greathouse J.A., Zhang X., Nenoff T.M. (2015). Enhanced O_2_ selectivity versus N_2_ by partial metal substitution in Cu-BTC. Chem. Mater..

[66] Wu Y., Liu D., Chen H., Qian Y., Xi H., Xia Q. (2015). Enhancement effect of lithium-doping functionalization on methanol adsorption in copper-based metal-organic framework. Chem. Eng. Sci..

[67] Wu Y., Chen H., Xiao J., Liu D., Liu Z., Qian Y., Xi H. (2015). Adsorptive Separation of Methanol–Acetone on Isostructural Series of Metal–Organic Frameworks M-BTC (M = Ti, Fe, Cu, Co, Ru, Mo): A Computational Study of Adsorption Mechanisms and Metal-Substitution Impacts. ACS Appl. Mater. Interfaces.

[68] Du M., Li L., Li M., Si R. (2016). Adsorption mechanism on metal organic frameworks of Cu-BTC, Fe-BTC and ZIF-8 for CO_2_ capture investigated by X-ray absorption fine structure. RSC Adv..

[69] Wang H., Qu Z.G., Zhang W., Yu Q.N., He Y.L. (2016). Experimental and numerical study of CO_2_ adsorption on copper benzene-1,3,5-tricarboxylate (Cu-BTC) metal organic framework. Int. J. Heat Mass Transf..

[70] Liu Y., Liu S., Gonçalves A.A., Jaroniec M. (2018). Effect of metal–ligand ratio on the CO_2_ adsorption properties of Cu–BTC metal–organic frameworks. RSC Adv..

[71] Liu Y., Ghimire P., Jaroniec M. (2019). Copper benzene-1,3,5-tricarboxylate (Cu-BTC) metal-organic framework (MOF) and porous carbon composites as efficient carbon dioxide adsorbents. J. Colloid Interface Sci..

[72] Salehi S., Anbia M., Razavi F. (2020). Improving CO_2_/CH_4_ and CO_2_/N_2_ adsorptive selectivity of Cu-BTC and MOF-derived nanoporous carbon by modification with nitrogen-containing groups. Environ. Prog. Sustain. Energy.

[73] Rey R., Møller K.B., Hynes J.T. (2002). Hydrogen bond dynamics in water and ultrafast infrared spectroscopy. J. Phys. Chem. A.

[74] Gentile F.S., Pannico M., Causà M., Mensitieri G., Di Palma G., Scherillo G., Musto P. (2020). Metal defects in HKUST-1 MOF revealed by vibrational spectroscopy: A combined quantum mechanical and experimental study. J. Mater. Chem. A.

[75] Nijem N., Canepa P., Kong L., Wu H., Li J., Thonhauser T., Chabal Y.J. (2012). Spectroscopic characterization of van der Waals interactions in a metal organic framework with unsaturated metal centers: MOF-74–Mg. J. Phys. Condens. Matter.

[76] Supronowicz B., Mavrandonakis A., Heine T. (2013). Interaction of small gases with the unsaturated metal centers of the HKUST-1 metal organic framework. J. Phys. Chem. C.

[77] Salazar J., Weber G., Simon J., Bezverkhyy I., Bellat J. (2015). Characterization of Adsorbed Water in MIL-53 (Al) by FTIR Spectroscopy and Ab-Initio Calculations. J. Chem. Phys..

[78] Rieth A.J., Hunter K.M., Dincă M., Paesani F. (2019). Hydrogen Bonding Structure of Confined Water Templated by a Metal-Organic Framework with Open Metal Sites. Nat. Commun..

[79] Gong Y., Xu Y., Zhou Y., Li C., Liu X., Niu L., Huang Y., Zhang X., Sun C.Q. (2017). Hydrogen Bond Network Relaxation Resolved by Alcohol Hydration (Methanol, Ethanol, and Glycerol). J. Raman Spectrosc..

[80] Arayachukiat S., Kongtes C., Barthel A., Vummaleti S.V., Poater A., Wannakao S., Cavallo L., D’Elia V. (2017). Ascorbic Acid as a Bifunctional Hydrogen Bond Donor for the Synthesis of Cyclic Carbonates from CO_2_ under Ambient Conditions. ACS Sustain. Chem. Eng..

[81] Munoz-Santiburcio D., Marx D. (2017). Chemistry in Nanoconfined Water. Chem. Sci..

[82] Wang H., Wagner J.C., Chen W., Wang C., Xiong W. (2020). Spatially Dependent H-Bond Dynamics at Interfaces of Water/Biomimetic Self-Assembled Lattice Materials. Proc. Natl. Acad. Sci. USA.

[83] Li J., Chi Z., Qin R., Yan L., Lin X., Hu M., Shan G., Chen H., Weng Y.-X. (2020). Hydrogen Bond Interaction Promotes Flash Energy Transport at MXene-Solvent Interface. J. Phys. Chem. C.

[84] He F., Wang Q., Hu C., He W., Luo X., Huang L., Gao D., Bi J., Wang X., Zou G. (2018). Centrosymmetric (NH4)2SbCl(SO4)2 and Non-Centrosymmetric (NH_4_)SbCl_2_(SO_4_): Synergistic Effect of Hydrogen-Bonding Interactions and Lone-Pair Cations on the Framework Structures and Macroscopic Centricities. Cryst. Growth Des..

[85] Singha A., Dhar P., Roy A. (2005). A nondestructive tool for nanomaterials: Raman and photoluminescence spectroscopy. Am. J. Phys..

[86] Bonino F., Chavan S., Vitillo J.G., Groppo E., Agostini G., Lamberti C., Dietzel P.D.C., Prestipino C., Bordiga S. (2008). Local Structure of CPO-27-Ni Metallorganic Framework upon Dehydration and Coordination of NO. Chem. Mater..

[87] Tan K., Nijem N., Canepa P., Gong Q., Li J., Thonhauser T., Chabal Y.J. (2012). Stability and hydrolyzation of metal organic frameworks with paddle-wheel SBUs upon hydration. Chem. Mater..

[88] Todaro M., Alessi A., Sciortino L., Agnello S., Cannas M., Gelardi F.M., Buscarino G. (2016). Investigation by Raman spectroscopy of the decomposition process of HKUST-1 upon exposure to air. J. Spectrosc..

[89] Martra G., Gianotti E., Coluccia S. (2008). The application of UV–visible–NIR spectroscopy to oxides. Metal Oxide Catalysis.

[90] Schoonheydt R.A. (2010). UV-VIS-NIR spectroscopy and microscopy of heterogeneous catalysts. Chem. Soc. Rev..

[91] Nilsson A. (2002). Applications of core level spectroscopy to adsorbates. J. Electron Spectrosc. Relat. Phenom..

[92] Garino C., Borfecchia E., Gobetto R., van Bokhoven J.A., Lamberti C. (2014). Determination of the electronic and structural configuration of coordination compounds by synchrotron-radiation techniques. Coord. Chem. Rev..

[93] Wang S., Yang Q., Zhong C. (2008). Adsorption and separation of binary mixtures in a metal-organic framework Cu-BTC: A computational study. Sep. Purif. Technol..

[94] Gutiérrez-Sevillano J.J., Calero S., Krishna R. (2015). Selective adsorption of water from mixtures with 1-alcohols by exploitation of molecular packing effects in CuBTC. J Phys. Chem. C.

[95] Grimme S. (2011). Density functional Theory with London Ddispersion Corrections. Wiley Interdiscip. Rev. Comput. Mol. Sci..

[96] St.Petkov P., Vayssilov G.N., Liu J., Shekhah O., Wang Y., Woll C., Heine T. (2012). Defects in MOFs: A Thorough Characterization. ChemPhysChem.

[97] Pidko E.A., Hensen E.J.M., Llabres i Xamena F.X., Gascon J. (2013). Computational Approach to Chemical Reactivity of MOFs. Metal Organic Frameworks as Heterogeneous Catalysts.

[98] Ptak M., Dziuk B., Stefanska D., Hermanowicz K. (2019). The Structural, Phonon and Optical Properties of [CH_3_NH_3_] M_0.5_Cr_x_Al_0.5-x_(HCOO)_3_ (M = Na, K; x = 0, 0.025, 0.5) Metal Organic Framework Perovskites for Luminescence Thermometry. Phys. Chem. Chem. Phys..

[99] Sips R. (1948). On the Structure of a Catalyst Surface. J. Chem. Phys..

[100] Do D.D. (1998). Adsorption Analysis: Equilibria and Kinetics.

[101] Pierini G., Viola A., Malara C. (1991). Correlation, Analysis and Prediction of Adsorption Equilibria.

[102] Richard M.A., Bénard P., Chahine R. (2009). Gas adsorption process in activated carbon over a wide temperature range above the critical point. Part 1: Modified Dubinin-Astakhov model. Adsorption.

[103] Kaur R., Kaur A., Umar A., Anderson W.A., Kansal S.K. (2019). Metal organic framework (MOF) porous octahedral nanocrystals of Cu-BTC: Synthesis, properties and enhanced adsorption properties. Mater. Res. Bull..

[104] Wang J., Wolf R.M., Caldwell J.W., Kollman P.A., Case D.A. (2004). Development and Testing of a General Amber Force Field. J. Comput. Chem..

[105] Rappé A.K., Casewit C.J., Colwell K., Goddard W.A., Skiff W.M. (1992). UFF, a Full Periodic Table Force Field for Molecular Mechanics and Molecular Dynamics Simulations. J. Am. Chem. Soc..

[106] Addicoat M.A., Vankova N., Akter I.F., Heine T. (2014). Extension of the Universal Force Field to Metal-Organic Frameworks. J. Chem. Theory Comput..

[107] Bureekaew S., Amirjalayer S., Tafipolsky M., Spickermann C., Roy T.K., Schmid R. (2013). MOF-FF–A Flexible First-Principles Derived Force Field for Metal-Organic Frameworks. Phys. Status Solidi B.

[108] Vanduyfhuys L., Vandenbrande S., Verstraelen T., Schmid R., Waroquier M., Van Speybroeck V. (2015). QuickFF: A Program for a Quick and Easy Derivation of Force Fields for Metal-Organic Frameworks from Ab Initio Input. J. Comput. Chem..

[109] Brandani S. (2019). The Rigid Adsorbent Lattice Fluid Model for Pure and Mixed Gas Adsorption. AIChE J..

[110] Verbraeken M.C., Brandani S. (2020). A priori prediction of type I and type V isotherms by the rigid adsorbent lattice fluid. Adsorption.

[111] Verbraeken M.C., Brandani S. (2019). Predictions of stepped isotherms in breathing adsorbents by the rigid adsorbent lattice fluid. J. Phys. Chem. C.

[112] Sanchez I.C., Lacombe R.H. (1976). An Elementary Molecular Theory of Classical Fluids. Pure fluids. J. Phys. Chem..

[113] Lacombe R.H., Sanchez I.C. (1976). Statistical Thermodynamics of Fluid Mixtures. J. Phys. Chem..

[114] Sanchez I.C., Lacombe R.H. (1978). Statistical Thermodynamics of Polymer Solutions. Macromolecules.

[115] Doghieri F., Sarti G.C. (1996). Nonequilibrium Lattice Fluids: A Predictive Model for the Solubility in Glassy Polymers. Macromolecules.

[116] Sarti G.C., Doghieri F. (1998). Predictions of the solubility of gases in glassy polymers based on the NELF model. Chem. Eng. Sci..

[117] Doghieri F., Canova M., Sarti G.C., Freeman B.D., Pinnau I. (1999). Polymer Membranes for Gas and Vapor Separation.

[118] Coleman B.D., Gurtin M.E. (1967). Thermodynamics with Internal State Variables. J. Chem. Phys..

[119] Astarita G. (1989). Thermodynamics: An Advanced Textbook for Chemical Engineers.

[120] De Donder T., Van Rysselberghe P. (1936). Thermodynamic Theory of Affinity.

[121] Brandani S., Mangano E., Sarkisov L. (2016). Net, excess and absolute adsorption and adsorption of helium. Adsorption.

[122] Neau E. (2002). A consistent method for phase equilibrium calculation using the Sanchez–Lacombe lattice–fluid equation-of-state. Fluid Phase Equilib..

[123] Nicolas C., Neau E., Meradjiac S., Raspoac I. (2005). The Sanchez-Lacombe lattice fluid model for the modelling of solids in supercritical fluids. Fluid Phase Equilib..

[124] Von Konigslow K., Park C.B., Thompson R.B. (2017). Polymeric foaming predictions from the Sanchez-Lacombe equation of state: Application to polypropylene-carbon dioxide mixtures. Phys. Rev. Appl..

[125] Von Konigslow K. (2017). An Off-Lattice Derivation and Thermodynamic Consistency Consideration for the Sanchez-Lacombe Equation of State. Ph.D. Thesis.

[126] Von Konigslow K., Park C.B., Thompson R.B. (2018). Application of a constant hole volume Sanchez-Lacombe equation of state to mixtures relevant to polymeric foaming. Soft Matter.

[127] Mensitieri G., Scherillo G., Panayiotou C., Musto P. (2020). Towards a predictive thermodynamic description of sorption processes in polymers: The synergy between theoretical EoS models and vibrational spectroscopy. Mater. Sci. Eng. R.

[128] Baldanza A., Loianno V., Scherillo G., Panayiotou C., Mensitieri G. (2022). On the Thermodynamic Consistency of Non-Random Hydrogen Bonding Lattice-Fluid Model for Multicomponent Mixtures. Fluid Phase Equilib..

[129] Panayiotou C., Pantoula M., Stefanis E., Tsivintzelis I., Economou I.G. (2004). Nonrandom Hydrogen-Bonding Model of Fluids and Their Mixtures. 1. Pure Fluids. Ind. Eng. Chem. Res..

[130] Panayiotou C., Tsivintzelis I., Economou I.G. (2007). Nonrandom Hydrogen-Bonding Model of Fluids and Their Mixtures. 2. Multicomponent Mixtures. Ind. Eng. Chem. Res..

[131] De Hoff R. (2006). Thermodynamics in Materials Science.

[132] Bird R.B., Klingenberg D.J. (2013). Multicomponent diffusion- a brief review. Adv. Water Resour..

[133] Scherillo G., Sanguigno L., Galizia M., Lavorgna M., Musto P., Mensitieri G. (2012). Non-equilibrium compressible lattice theory accounting for hydrogen bonding interactions: Modelling water sorption thermodynamics in fluorinated polyimides. Fluid Phase Equilib..

[134] Panayiotou C., Sanchez I.C. (1991). Hydrogen bonding in fluids: An equation-of-state approach. J. Phys. Chem..

[135] Veytsman B.A. (1990). Are Lattice Models Valid for Fluids with Hydrogen Bonds?. J. Phys. Chem..

[136] Brandani S., Mangano E., Santori G. (2022). Water adsorption on AQSOA-FAM Z02 beads. J. Chem. Eng. Data.

[137] Musto P., La Manna P., Pannico M., Mensitieri G., Gargiulo N., Caputo D. (2018). Molecular interactions of CO_2_ with the CuBTC metal organic framework: An FTIR study based on two-dimensional correlation spectroscopy. J. Mol. Struct..

[138] Cunliffe-Jones D.B. (1969). Perturbation of some vibrational bands in solution. Spectrochim. Acta A.

[139] Kazarian S.G., Vincent M.F., Bright F.V., Liotta C.L., Eckert C.A. (1996). Specific Intermolecular Interaction of Carbon Dioxide with Polymers. J. Am. Chem. Soc..

[140] Mensitieri G., Scherillo G., La Manna P., Musto P. (2019). Sorption thermodynamics of CO_2_, H_2_O and CH_3_OH in a glassy polyetherimide: A molecular perspective. Membranes.

[141] Musto P., Galizia M., Pannico M., Scherillo G., Mensitieri G. (2014). Time-resolved Fourier transform infrared spectroscopy, gravimetry, and thermodynamic modeling for a molecular level description of water sorption in poly (ε-caprolactone). J. Phys. Chem. B.

[142] Galizia M., La Manna P., Pannico M., Mensitieri G., Musto P. (2014). Methanol diffusion in polyimides: A molecular description. Polymer.

[143] Noda I., Ozaki Y. (2004). Two-Dimensional Correlation Spectroscopy: Applications in Vibrational and Optical Spectroscopy.

[144] Grajciar L., Nachtigall P., Bludsky O., Rubes M. (2015). Accurate ab initio description of adsorption on coordinatively unsaturated Cu^2+^ and Fe^3+^ sites in MOFs. J. Chem. Theory Comput..

[145] Mehio N., Dai S., Jiang D. (2014). Quantum Mechanical Basis for Kinetic Diameters of Small Gaseous Molecules. J. Phys. Chem. A.

[146] Hu H., Zhu J., Yang F., Chen Z., Deng M., Weng L., Ling Y., Zhou Y. (2019). A robust etb-type metal–organic framewrk showing polarity-exclusive adsorption of acetone over methanol for their azeotropic mixture. Chem. Commun..

[147] ALOthman Z.A. (2012). A review: Fundamental aspects of silicate mesoporous materials. Materials.

[148] Bingel L.W., Chen A., Agrawal M., Sholl D.S. (2020). Experimentally verified alcohol adsorption isotherms in nanoporous materials from literature meta-analysis. J. Chem. Eng. Data.

[149] Lemmon E.W., Bell I.H., Huber M.L., McLinden M.O., Linstrom P.J., Mallard W.G. (1998). Thermophysical Properties of Fluid Systems. NIST Chemistry WebBook, NIST Standard Reference Database Number 69.

[150] Decker G.E., Bloch E.D. (2021). Using Helium Pycnometry to Study the Apparent Densities of Metal–Organic Frameworks. ACS Appl. Mater. Interfaces.

