# Supplementary material for: Survey on Adsorption of Low Molecular Weight Compounds in Cu-BTC Metal–Organic Framework: Experimental Results and Thermodynamic Modeling
Source: Int J Mol Sci. 2022 Aug 20;23(16):9406. doi: 10.3390/ijms23169406 (PMC9409301; doi:10.3390/ijms23169406)
Supplement: Supplementary file 1 [file ijms-23-09406-s001.zip › ijms-1880259-supplementary.pdf]

Supplementary materials

Survey on adsorption of low molecular weight compounds in Cu-BTC Metal-Organic-Framework: experimental results and thermodynamic modelling

Antonio Baldanza, Domenico Mallamace, Giuseppe Mensitieri, Cosimo Brondi, Pellegrino Musto and Giuseppe Scherillo

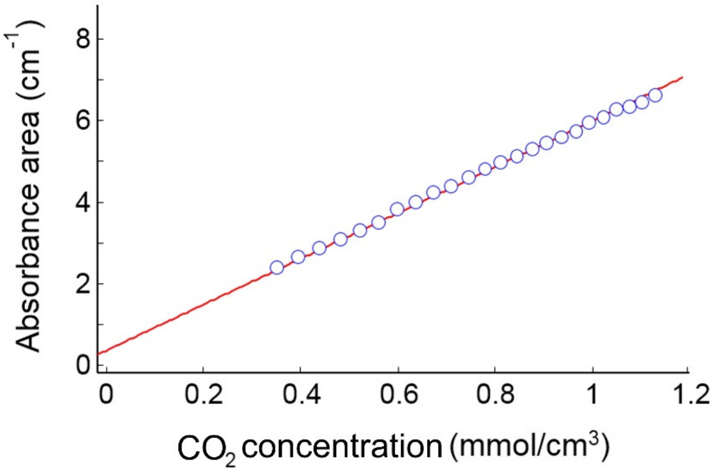

Figure S1. Correlation between spectroscopic and volumetric data relative to a CO_2 sorption isotherm at 35°C.

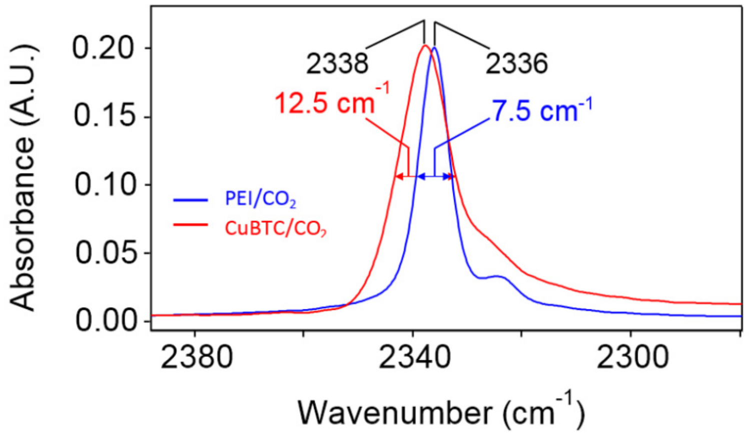

Figure S2. Bandshape of CO_2 sorbed in polyetherimide (PEI) and in CuBTC. Normalization with respect to the PEI system has been made to facilitate the comparison.

Table S1. Optimized fitting parameters of RALF model

T_S^* (K)	986
P_S^* (MPa)	2754
k_{CO_2-S}	0.2571
k_{CH_4-S}	0.1758
k_{N_2-S}	0.1287
k_{O_2-S}	0.3607
$k^{I}_{CH_3OH-S}$	-0.0071
$k^{II}_{CH_3OH-S}$	0.3210
ζ_{CO_2-S}	0.3151
ζ_{CH_4-S}	0.6088
ζ_{N_2-S}	0.4077
ζ_{O_2-S}	0.0731
$\zeta^{I}_{CH_3OH-S}$	0.0002
$\zeta^{II}_{CH_3OH-S}$	0.0031
ω_I	0.5467
$f_{V,I}$	0.6437

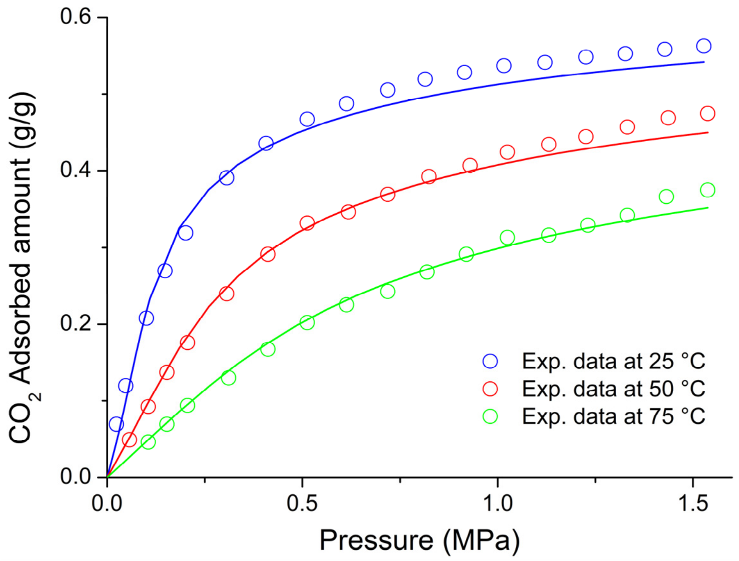

Figure S3. Comparison between experimental data and simultaneous optimized model fitting (solid lines).

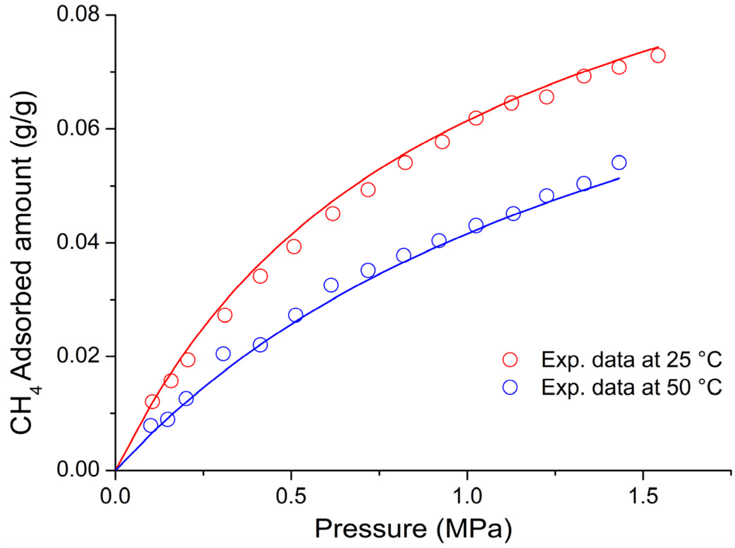

Figure S4. Comparison between experimental data and simultaneous optimized model fitting (solid lines).

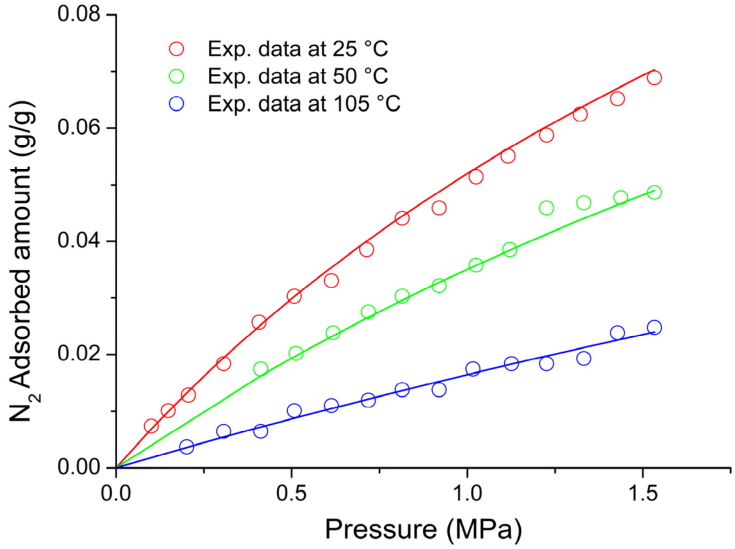

Figure S5. Comparison between experimental data and simultaneous optimized model fitting (solid lines).

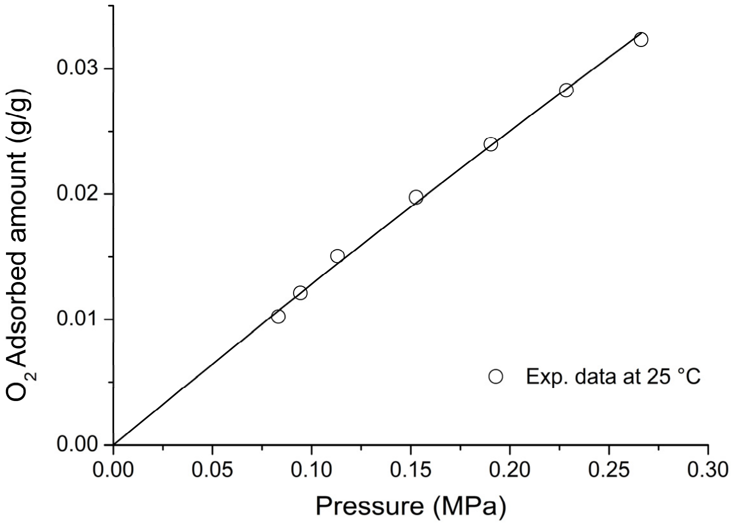

Figure S6. Comparison between experimental data and simultaneous optimized model fitting (solid lines).

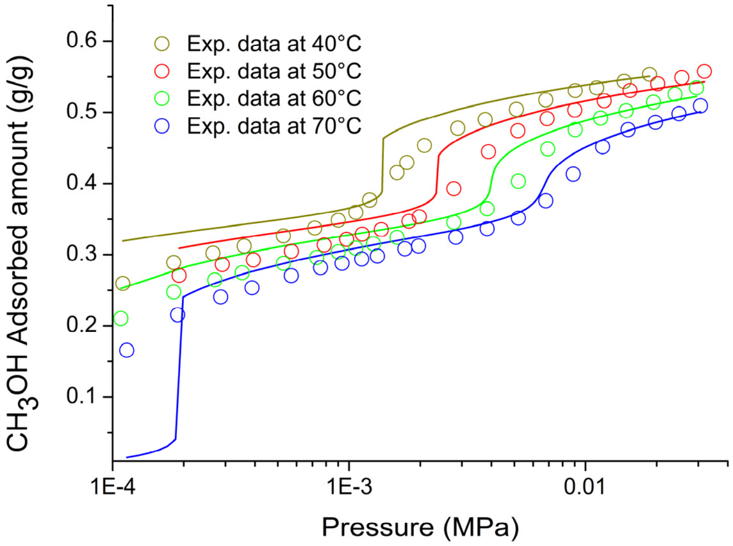

Figure S7. Comparison between experimental data and simultaneous optimized model fitting (solid lines).
